# Computational Modeling of Cobalt-Based Water Oxidation: Current Status and Future Challenges

**DOI:** 10.3389/fchem.2018.00100

**Published:** 2018-04-18

**Authors:** Mauro Schilling, Sandra Luber

**Affiliations:** Department of Chemistry, University of Zürich, Zurich, Switzerland

**Keywords:** water oxidation, catalysis, computational chemistry, density functional theory, reaction mechanism

## Abstract

A lot of effort is nowadays put into the development of novel water oxidation catalysts. In this context, mechanistic studies are crucial in order to elucidate the reaction mechanisms governing this complex process, new design paradigms and strategies how to improve the stability and efficiency of those catalysts. This review is focused on recent theoretical mechanistic studies in the field of homogeneous cobalt-based water oxidation catalysts. In the first part, computational methodologies and protocols are summarized and evaluated on the basis of their applicability toward real catalytic or smaller model systems, whereby special emphasis is laid on the choice of an appropriate model system. In the second part, an overview of mechanistic studies is presented, from which conceptual guidelines are drawn on how to approach novel studies of catalysts and how to further develop the field of computational modeling of water oxidation reactions.

## 1. Introduction

In the past decade, artificial water splitting has become a hot topic in research on renewable energy sources. A crucial step in this process is the oxidation of water to molecular oxygen. There are numerous catalysts which have been shown to facilitate this reaction, both of homogeneous and heterogeneous nature. Even though some of them show outstanding catalytic performance, we still lack fundamental understanding of the catalytic process. The latter is crucial in order to systematically improve those catalysts in terms of their catalytic performance and long term stability. The complexity of such systems often makes experimental investigations of catalytic intermediates a very tedious task. Computational approaches provide additional insight, by either helping to explain experimental findings or by simulations of systems and processes for which no experimental data is available. In the following, we will review some of the recent theoretical studies based on homogeneous transition metals complexes used for water oxidation with emphasis on cobalt-based catalysts. The latter has not been the most commonly used metal for artificial water oxidation catalysts (WOCs). However, it has become more and more popular in recent years, and when it comes to mimicking the cuboidal structure of nature's oxygen evolution cluster (OEC), cobalt-based WOCs turned out to be more active compared to their manganese-based counterparts (Evangelisti et al., 2013, 2015).

We focus our attention not only on the outcome of the studies at hand, but also on the applied methodology. This review contains two main sections: In the first one we discuss the currently accepted mechanisms for water oxidation. We will in particular discuss the choice of the model system and computational approaches applied to tackle specific questions associated with those mechanisms. In the second section, we give the reader a brief overview on the current state of field by discussing the most important contributions with respect to homogeneous Co-based WOCs.

## 2. Water oxidation mechanisms

From a chemical point of view the water oxidation is a straight forward process (see Equation 1) where the oxygen atoms of two water molecules are oxidized, while the protons and electrons are released, and the two oxygen atoms combine to form molecular oxygen:

(1) $${2\text{H}}_{2}\text{O}\to 4{\text{H}}^{+}+{\text{O}}_{2}+4{\text{e}}^{-}$$

Even if enough energy is supplied to the system to overcome the thermodynamic barrier (i.e., 4.92 eV at standard conditions) the reaction still does not occur spontaneously. Only in the presence of a suitable catalyst reasonable turnover numbers can be achieved. But which properties should such a catalyst have? An intuitive definition of an ideal catalyst was recently given by Balcells (2016), who states that an ideal catalyst has to fulfill the following four criteria: (1) high activity under mild conditions (neutral pH, room temperature, atmospheric pressure); (2) longterm stability in order to achieve high turnover numbers; further it should be easy to recycle the catalyst after it has lost its activity; (3) cheap and “green”, i.e., friendly toward the environment; this is mostly true for catalysts containing first row transition metals such as cobalt discussed later on. However, the organic ligand framework might still be a health risk for certain animals or plants; (4) modular, the catalyst should be easy to modify and to immobilize or couple to other catalytically active species. Not all of those criteria are easily targetable by computational studies, however in particular (1) offers various opportunities where theoretical studies can greatly enhance the fundamental understanding of the catalytic processes at hand.

The basic questions behind (1) are in general: What is the catalytic mechanism, what is the rate-limiting step, and how can the catalytic performance possibly be improved?

In order to elucidate these issues we have to decompose the reaction (Equation 1) into its elementary reactions. In principle there are four oxidations [electron transfers (ETs)], four deprotonations [proton transfers (PTs)], the oxygen-oxygen bond formation (will be discussed later in more detail), the release of molecular oxygen [O_2_ dissociation (O2DI)] as well as the association of a water molecule to regenerate the catalyst [aqua association (AQAS)]. Some of those elementary reactions might occur combined such as proton coupled electron transfers (PCETs); likewise, the release of molecular oxygen and the regeneration might happen in a concerted fashion.

**Figure 1:**
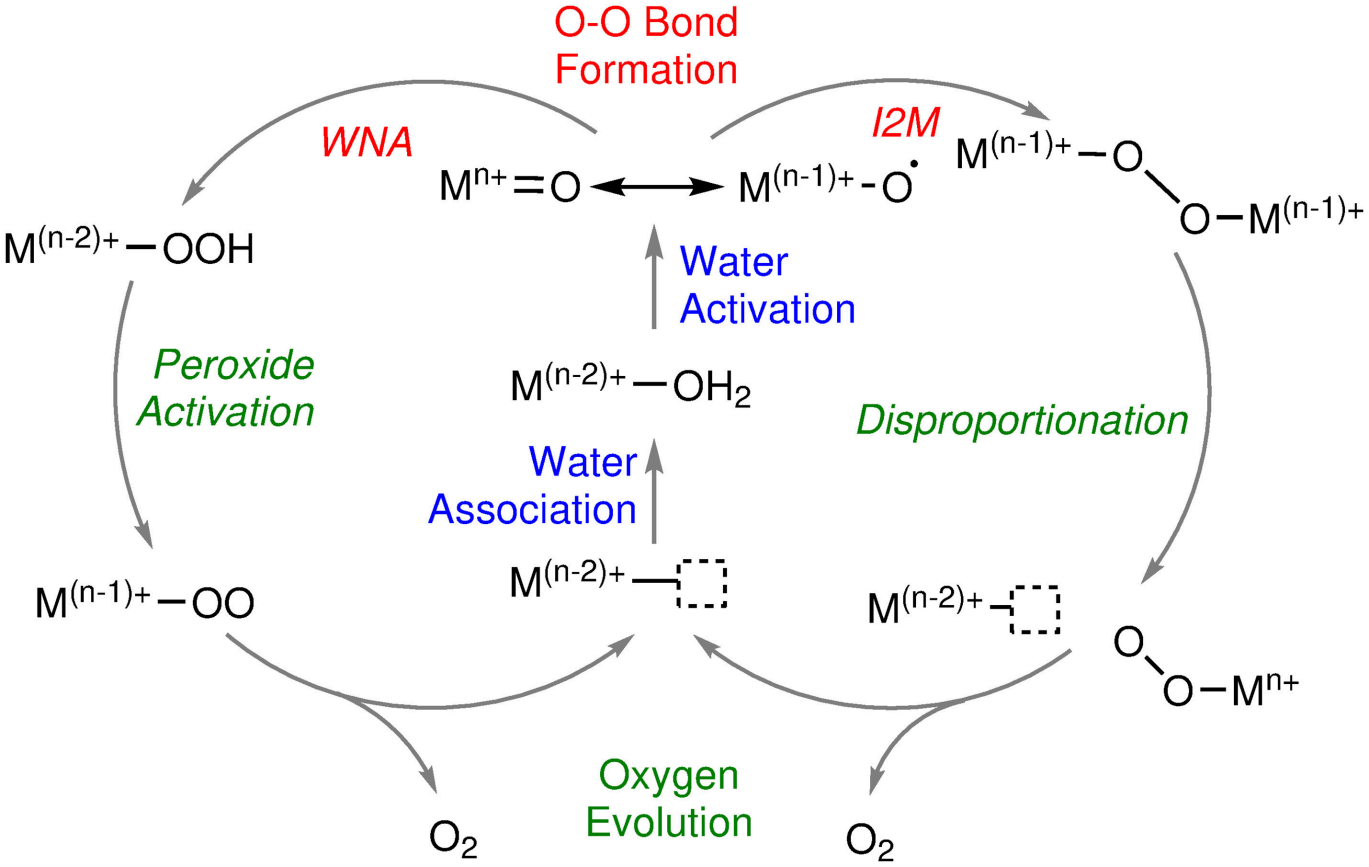
Schematic overview of water oxidation mechanisms. M represents any redox active transition metal, n is the highest formal oxidation state reached by the metal center during catalysis (Schematic drawing inspired by Shaffer et al., 2017).

In general, all water oxidation mechanisms can be categorized into three phases: in the first stage of the catalytic cycle, the catalyst undergoes several oxidations (ETs) and deprotonations (PTs) to reach a high oxidized state that contains an oxyl / oxo ligand (see Figure 1). The latter is involved in the oxygen-oxygen bond formation, either by a water nucleophilic attack (WNA) or by a radical-coupling (RC) reaction. The latter is sometimes also referred to as interaction of two metal oxo (I2M) species. For this review, we will use the more general term RC. After the bond formation, further oxidation (ET) and deprotonation (PT) reactions might take place before in the last stage of the mechanism molecular oxygen is released (O2DI), and the initial state of catalyst is regenerated by coordination of a novel substrate (AQAS).

**Figure 2:**
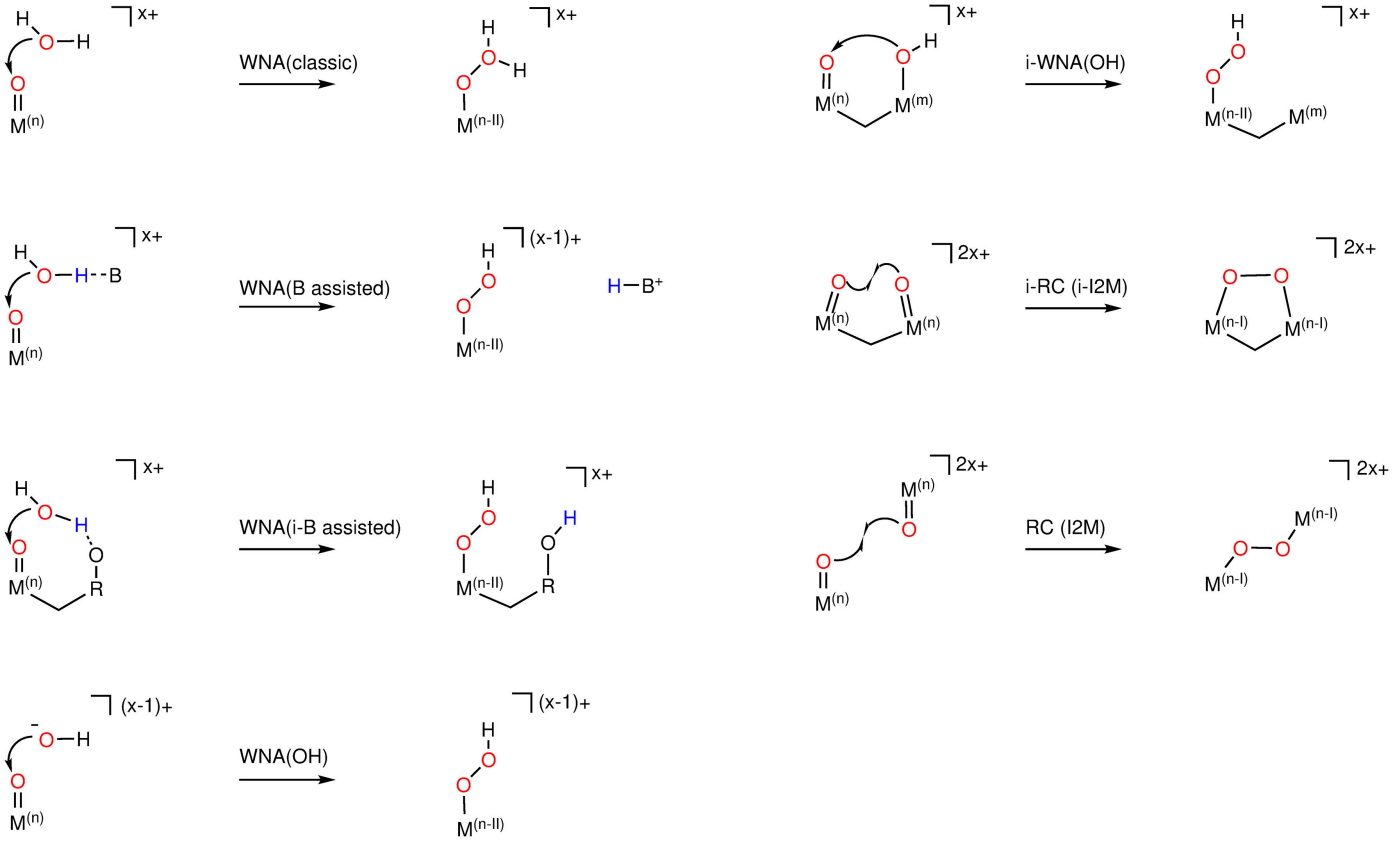
O−O bond formation mechanisms, where M^*n*/*m*^ represents any redox-active metal center in oxidation state (+n or +m). B is a general base in solution, and R is any residue including other transition metals. The overall charge of the association complex as well as of the product is given to highlight charge conservation. Note: Conceptually the product of a WNA, assisted by an intramolecular base [WNA(i-B assisted)] is a possible intermediate of the so-called “bifunctional mechanism” (see section 3.2), assuming the O−O bond formation and the PCET are not concerted reaction.

The exact order of those building blocks is dependent on the nature of the catalyst, i.e., the transition metal center(s) and associated oxidation state(s) as well as ligands and environment. There are also different flavors of the oxygen-oxygen bond formation reactions. For example, for the WNA, either a water molecule (WNA) or a hydroxide (WNA(OH)) can be the nucleophile which attacks the oxo / oxyl species in an intra- (i-WNA) or intermolecular reaction. Similarly, the RC mechanism can either be intra- (i-RC) or intermolecular (see Figure 2).

In the next section we will focus on the choice of appropriate computational settings and model systems picking up questions arising from points mentioned above.

## 3. Choosing a model system and an appropriate computational setting

Often it is a straight forward process to propose a water oxidation mechanism for a novel catalyst. However, experimentally it is challenging to get hands on intermediates which might help to elucidate details of the catalytic cycle. Here, computational chemistry is an essential tool to obtain an in-depth understanding on the electronic structure of the catalyst and possible intermediates thereof during catalysis. The work-horse of computational chemistry, density functional theory (DFT), usually is a suitable choice. Yet, in the course of this review we try to point out some limitations when it comes to modeling WOCs.

In order to get an overview of possible intermediates involved in the catalytic cycle, the first step is to calculate their relative stability in terms of electronic or free energies. Starting from those, a selection of structures can be made which are within a reasonable energy range, and can serve as building blocks for the full catalytic cycle. However, it is not enough to solely propose possible intermediates based on their relative stability, also their physical properties have to be in agreement with observable quantities such as spectroscopic data, reduction potentials (*E*_*red*_) and *pK*_*A*_ values describing their reactivity under reaction conditions. For a broad overview on which properties of WOCs are accessible by DFT we refer the reader to a review by Mavros et al. (2014). For an overview in terms of reaction barriers and mechanisms we refer to the work of Balcells (2016) and Liao and Siegbahn (2017). As pointed out by these authors, when modeling the thermodynamics and reaction barriers it boils down to the choice of a reasonable model system which is able to capture both solvation effects as well as spin-state energetics. The latter will be discussed in the following in the context of solvation effects, reaction barriers and the oxo/oxyl species.

### 3.1. Solvation effects

WOCs are operable in aqueous solutions whereby water is not only the solvent but also the substrate. Description of solvation effects hence becomes a crucial point when modeling properties such as *E*_*red*_ and *pK*_*A*_ values or reaction barriers. In principle two different approaches exist on how to model the aqueous environment of the catalyst. On the one hand, there are implicit (or continuum) solvent models such as the conductor-like screening model (COSMO), the polarizable continuum model (PCM), and others, where the interactions of the individual solvent molecules are averaged and represented as a dielectric continuum which interacts with the solute (Tomasi et al., 2007). Directed interactions such as hydrogen-bonds are thus not well described (Cramer and Truhlar, 1999; Tomasi, 2004; Skyner et al., 2015). Implicit solvation models suffer from an additional drawback, namely their (empirical) parameterization. In general such models are parameterized for a set of small organic molecules with a neutral charge computed at a rather low level of theory (i.e., small basis set and generalized gradient approximation (GGA) functionals). As a consequence their application to more complex systems such as charged transition metal complexes might not be fully justified in all cases. Nevertheless, they have been successfully applied to such systems when relative rather than absolute solvation free energies were of interest (Baik and Friesner, 2002). Another important point one should keep in mind is that the parameterization does not allow for a systematic improvement of the solvation free energies when moving to a higher level of theory in the calculations (Ho et al., 2010). With the development of the conductor-like screening model for real solvents (COSMO-RS), important improvements to the existing methodology of implicit solvation models were made, which allowed to alleviate some of their limitations such as the description of hydrogen bonding (Klamt, 2011). On the other hand, there are explicit (or atomistic) solvent models where the solvent molecules are an essential part of the model system. The main drawback of models which include explicit solvent molecules is the prerequisite to know which solvent molecules are essential for a sophisticated description of the system under investigation. Selection and subsequent optimization of certain solvent configurations may thus strongly bias the outcome of calculations. Extensive configuration sampling e.g., by molecular dynamics (MD) is often necessary making such models computationally demanding. Both static implicit and explicit solvation models were routinely combined, in particular for the calculation of reaction barriers for O−O bond formation where inclusion of several explicit water molecules in addition to a solvent continuum model had turned out to be crucial to stabilize the transition state by hydrogen bonds (Gil-Sepulcre et al., 2017). Recently, Hodel et al. (2017) published a study investigating the influence of different solvation models on the thermodynamics of ligand exchange reactions at a cobalt-based WOC. Employing implicit (COSMO and COSMO-RS), static explicit (solvation shells were extracted from DFT-MD) and dynamic explicit (sampled configurations from DFT-MD) solvation models, electronic energy differences obtained by COSMO-RS were found to be in better agreement with the difference obtained from DFT-MD than the one obtained with COSMO, while the static explicit solvation (as expected) depends on the chosen configuration and can thus lead to ambiguous electronic energy differences. Besides the relative stabilities of intermediate species, also reaction barriers were calculated employing nudged-elastic-band (NEB) to a model with explicit solvation, and metadynamic calculations where the solvent was treated dynamically (see section 3.2 for more details). Due to inadequate and static explicit solvation, NEB calculations resulted in a vast overestimation of the electronic reaction barrier. By virtue of the large system size required for an appropriate explicit solvation, sampling by DFT-MD is very costly. Here, the quantum mechanics/molecular mechanics (QM/MM) methodology promises to be a cheaper alternative, where the system (e.g., the transition metal complex and the first solvation shell) is treated at a QM level of theory, and the remaining part is dealt with using classical mechanics. Further examples of the application of the different solvation approaches can be found in section 4.

No matter which solvation model is applied it still remains an idealized model of the “real” reaction mixture which is often much more complex than models cover nowadays. Besides counter-ions, there are buffer molecules (Evangelisti et al., 2015) and — in the case of chemical oxidation – sacrificial oxidizing agents as well as their reduced products. Those complex mixtures are not only challenging from a theoretical point of view, also experimentalists have to come up with advanced strategies to verify the integrity of the catalysts before, during and after catalysis. If and how those components affect the calculated properties has to be determined for each system individually. In particular, when modeling the O−O bond formation, the pH determines whether a water molecule or a hydroxide is the nucleophile. Further, solvated cations might help to stabilize the transition state by participating in the hydrogen bonding network affecting the local pH or by direct coordination of the formed hydroperoxo species as proposed by Bucci et al. (2016) for a mononuclear iridium catalyst. The role of the oxidant can be even more pronounced as in the case of peroxymonosulfate, which cannot only be involved in ET transfer reactions but rather transfer an oxygen of its peroxo group to the catalyst, which in turn becomes the substrate for the O−O bond formation (Khan et al., 2015). This incomplete list of possible interactions illustrates that the complexity of the “real” system might not be caught by oversimplified models.

### 3.2. Reaction barriers

In order to elucidate the reaction mechanism, knowledge about the thermodynamics and other properties (see section 3.4) is not enough. Further insight regarding the kinetics is required which can be obtained by calculating reaction barriers. In order to be able to compare them to experimental turn-over-frequencies (TOFs), identification of the rate-determining-step (RDS) is necessary. For water oxidation mechanisms one often assumes that the chemical steps, i.e., the oxygen-oxygen bond formation, the release of molecular oxygen or the association of water, are rate limiting. Among those reactions, the most critical is the oxygen-oxygen bond formation (Fan et al., 2016). In general the calculation of reaction barriers is a difficult task since, depending on the model system, the reaction coordinate might be of high dimensionality and therefore localization of a transition state becomes challenging. In the following we will discuss the WNA and RC mechanisms for the O−O bond formation in more detail and point out critical choices which have to be made in order to model such reactions. A WNA either by a water or hydroxide molecule is governed by the spatial accessibility and the electrophilicity of the oxo / oxyl ligand of the catalyst. When a water molecule acts as a nucleophile, it has become a paradigm to introduce an intramolecular base with an appropriate orientation to deprotonate the approaching water, thereby increasing its nucleophilicity (Dogutan et al., 2011). Lately, this concept has also been applied to model systems of heterogeneous catalysts by Frydendal et al. (2015) and Busch et al. (2016) where a hydrogen acceptor is placed next to oxo / oxyl ligand, which deprotonates the approaching nucleophile. In this way, the calculation of highly unstable hydroperoxo species (M−OOH) can be avoided since directly the superoxo species M−OO is formed. This mechanism is referred to as “bifunctional” (see Figure 2 - WNA(i-B assisted)). However, if it is the goal to calculate the barrier for the O−O bond formation, combination with an ET is not an option. From a conceptual point of view, there is no difference whether the superoxo species M−OO is formed by a classical WNA ([WNA-PCET]-PCET) or by a bifunctional WNA ([WNA-PT-PCET]-ET), the latter just includes an additional PT into the first transformation as indicated by the brackets. Please note, the brackets representing a concerted reaction in a chemical sense might be chosen for computational reasons only and are probably not always intuitive. So, if an intramolecular base enhances the nucleophilicity of H_2_O why is not OH^−^ used from the beginning? Besides the experimental conditions which often do not allow the existence of OH^−^ there is another problem, namely the Coulomb repulsion between OH^−^ and the oxo / oxyl species (discussed later in detail - see section 3.3) (Crandell et al., 2015).

**Figure 3:**
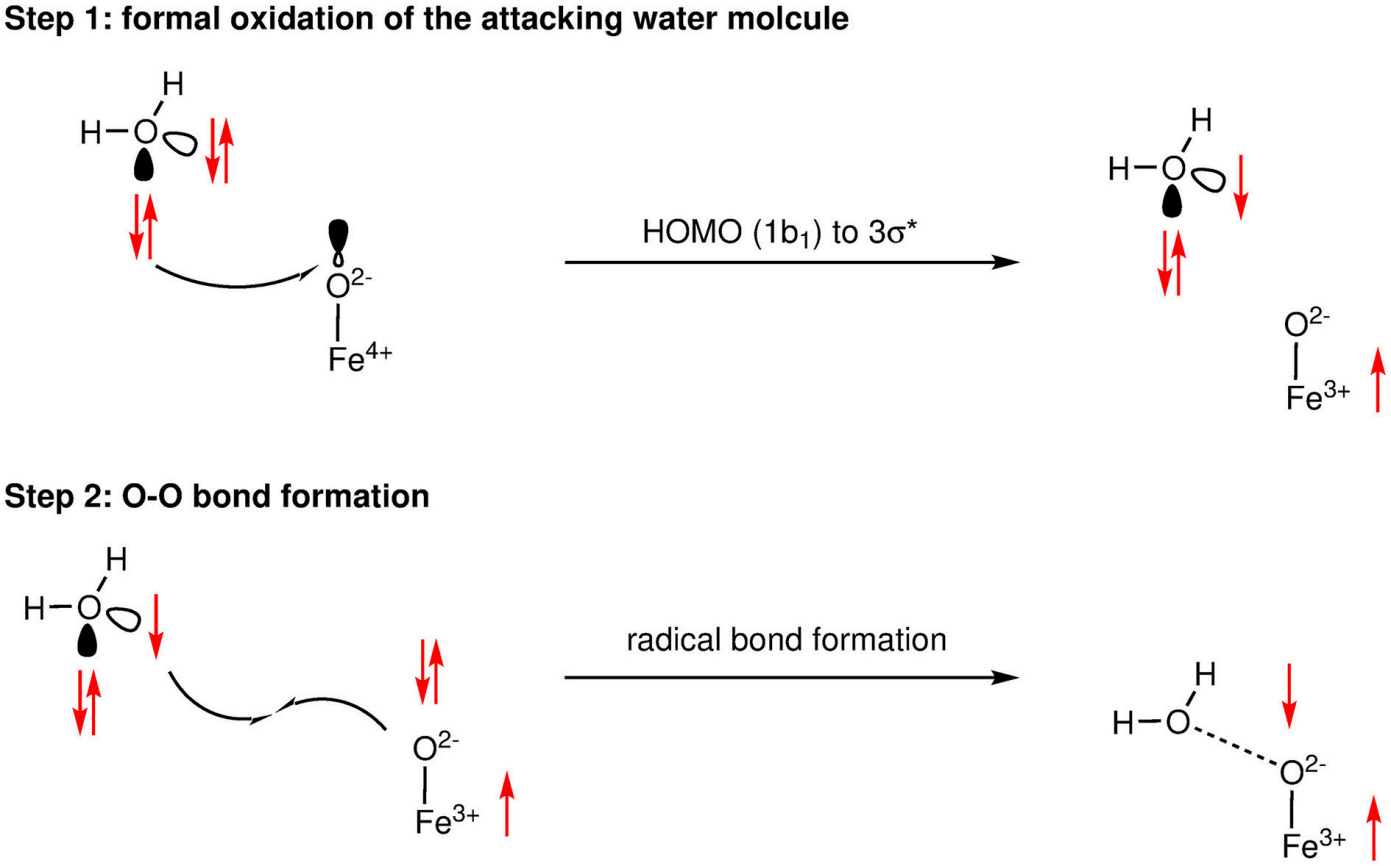
Schematic representation of the SET-WNA mechanism of the O−O bond formation as determined from AIMD simulations. Reprinted with permission from Bernasconi et al. (2017). Copyright 2018 American Chemical Society.

A critical choice in the calculation of the reaction barrier for the WNA mechanism is the solvation model. The use of an implicit solvation model is often not enough since the approaching nucleophile and transition state might require further stabilization by hydrogen bonding. Hence, it is common to include several water molecules into the transition state models (see Tong et al., 2011; Gil-Sepulcre et al., 2017). If the number of water molecules is increased this approach quickly reaches its limitations, namely when the degrees of freedom of the solvent and solute become so large that sampling is required. An example where this model was pushed toward its limit is the work by Hodel and Luber (2016a,b). Inclusion of the whole first solvation shell led to novel insight into the proton-transfer patters using climbing image-nudged elastic band (CI-NEB) calculations for the O−O bond formation. However, the obtained electronic energy differences from such static solvation models strongly depend on their initial geometries. Combining both ab initio molecular dynamics (AIMD) and NEB calculations (Mattioli et al., 2013) investigated water oxidation mechanisms by cobalt oxide clusters as models for heterogeneous Co-oxides. The next logical step to go beyond the static model of NEBs is sampling the whole configurational space of the solute and solvent. The metadynamic protocol first proposed by Laio and Parrinello (2002) offers the opportunity to sample the phase space with regards to observables which are indicative for the reaction. The latter was successfully applied to small model WOCs in explicit solvation (Vallés-Pardo et al., 2012; Piccinin et al., 2013). While metadynamics simulations give a more realistic picture of the reaction than static approaches and might allow further insight on how the solvent molecules participate in the O−O bond formation, they are still depended on the choice of the observables, the bias potential (collective variables), and the quality of the sampling, all of which make such simulations computationally very demanding and therefore de facto limit their application to small systems. Besides metadynamics, there are other protocols where AIMD simulations have been biased in order to model reaction barriers. Bernasconi et al. (2017) obtained activation barriers by applying the thermodynamic integration scheme where the constrained O−O bond of an Fe^IV^ = O complex was scanned in a discrete interval. Their approach allowed them to observe a step-wise mechanism for the WNA, where first the approaching H_2_O is oxidized by the transition metal complex, in a second step the O−O bond formation takes place between the two radicaloid species, followed by a deprotonation to form the well-known hydroperoxo species (see Figure 3). Unlike conventional water oxidation where molecular oxygen is the final product, the model catalysts discussed by Bernasconi et al. (2017) produce hydrogen peroxide. The same step-wise mechanism was earlier reported by Funes-Ardoiz et al. (2017) for a set of copper catalysts, they referred to it as a single electron transfer-water nucleophilic attack (SET-WNA).

As indicated earlier, the hydroperoxo species formed by a WNA is a rather unstable intermediate which might require further stabilization by solvent molecules or aqua ions. The RC mechanism completely avoids the formation of such an intermediate and thus is supposedly less dependent on external factors. However, it suffers from other complications, among them are the intermolecular interactions between the two oxo-moieties, such as π − π stacking of their ligands (dispersion) or the interaction with the solvent. Further, there is the question how the actual bond formation should be described, thereby in particular the spin state and its change during the reaction are of interest.

Recently, Fan et al. (2016) raised the important question, why the RC mechanism is not always favored over WNA mechanism since theoretically there should be no enthalpic barrier for such a reaction. In other words, the behavior of the potential energy as a function of the O−O distance should in principle be similar to the dissociation of molecular oxygen and hence be barrier free. Of course, for real systems such as the well-known family of Ru-bda (2,2′-bipyridine-6,6′-dicarboxylate) catalysts by *Sun* and coworkers (Duan et al., 2012b,a; Wang et al., 2012; Duan et al., 2013; Wang et al., 2014; Duan et al., 2015) other effects such as the relative positioning of the catalysts and/or the rearrangement of their ligand sphere might be associated with a significant energetic penalty which disfavors a RC mechanism. Those secondary interactions are governed by the nature of the axial ligands, which in the past have been the focus of both experimental and theoretical studies. For example, Fan et al. (2016) probed whether the electronic activation barriers for different substitution patterns on the axial ligands correlate with the electronic structure of the ligands (e.g., electron donating, electron withdrawing). However, the effect on the barriers appeared to be minimal. Therefore, they concluded that the formation of the encounter complex prior to the actual O−O bond formation is the energetically limiting factor. In a follow up study, they investigated the interaction of the solvent with the solute employing an empirical valence bond approach by Warshel and Weiss (1980) which is based on molecular mechanics simulations. They found that an explicit treatment of the solvent is required in order to facilitate π − π stacking of the axial ligands. Further, the oxo-species was found to be hydrophobic which can explain why the two monomers form a face-face encounter complex. For an overview on the current state of research about Ru-based WOCs, we point the reader's attention to a recent review by Shaffer et al. (2017).

Until now we primarily focused on secondary interactions which govern the formation of the encounter complex and not on the O−O bond formation *per se*. The latter is usually described as an open-shell singlet state (Nakano, 2017), where the two radicals have anti-parallel spin, description of which is difficult with single determinant methods such as DFT. The broken-symmetry (BS) approach by Yamaguchi et al. (1986) and Noodleman et al. (1988) often turned out to be a rather good approximation to the real multi-determinant problem. Further, a rigorous treatment by more accurate wave function based methods is mostly computationally not feasible for transition metal based WOCs. Since the early work of Yamaguchi et al. (1986) and Noodleman et al. (1988) alternative formulations have been proposed (Ferre et al., 2015). Taking all those considerations into account, nowadays scans of the O−O bond have become the standard tool to estimate reaction barriers. Thereby, the antiferromagnetic singlet state (open shell singlet) was often found to be the energetically most favorable state (Yang and Baik, 2004, 2006, 2008; Nyhlén et al., 2010). In general the geometries for the different spin states are slightly different which in turn also affects the energetics as well as the magnetic properties. However, for transition metal complexes those structural changes are often found to be negligible (Malrieu and Trinquier, 2012).

The discussion of electronic structure leads us directly to the next topic - namely how to accurately describe the metal-oxo species, which by now we have identified as the key intermediate for all water oxidation mechanisms.

### 3.3. Metal-oxo

Those species are not only interesting in the context of water oxidation, they also play an important role in various other processes, for instance as cofactors in cytochrome P450 enzymes which catalyze oxygen atom transfer reactions in biological systems. Such porphyrin-complexes have been studied in detail with a large variety of methods, reviewing of which is beyond the scope of the current work. We therefore focus on a few selected publications which are related to water oxidation.

**Figure 4:**
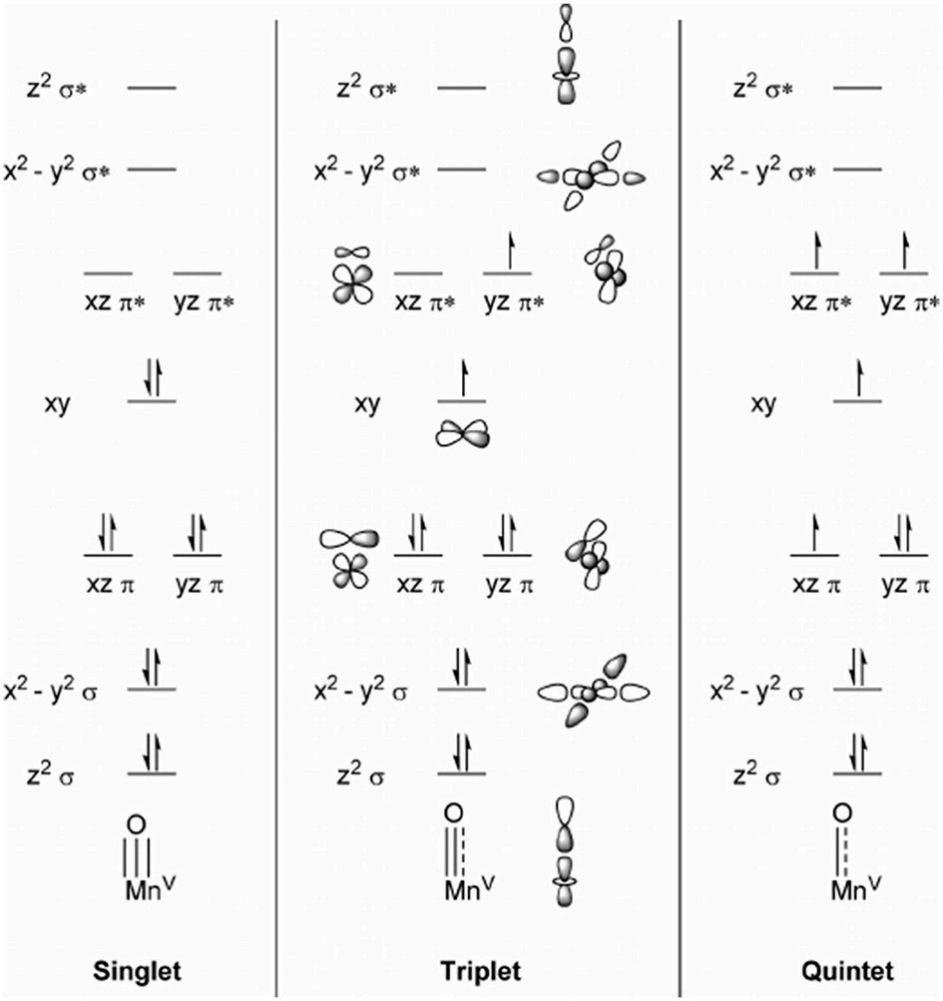
Simplified MO diagrams for different spin-states of Mn^V^ = O. Reprinted with permission from Ashley and Baik (2016). Copyright 2018 American Chemical Society.

Before we discuss the advantages and failures of different methods when describing metal-oxo species let us start from a purely chemical point of view. In literature metal-oxo species are often referred to as {M=O}^(n−2)+^ ↔ {M−O^•^}^(n−2)+^, but this simplified picture is somehow misleading since it describes the oxidation states of the individual components M^n+^ + O^2−^ and M^(n−1)+^ + O^•−^ prior to the actual bond formation (i.e., mixing of metal and ligand orbitals). A simplified picture of the molecular orbitals (MOs) of Mn^V^ = O is shown in Figure 4, where in an idealized ionic ligand field the π orbitals would be entirely localized on oxygen while the π^*^ orbitals would be localized on the metal center. However, in reality mixing between the 2p and 3d orbitals occurs and the radical is delocalized over the whole M−O bond (Venturinelli Jannuzzi et al., 2016). An accurate description of the latter is required in order to rationalize the O−O bond formation. But this is exactly where DFT is known to often fail, the calculation of spin state energetics (see Szalay et al., 2012; Roos et al., 2016, for a more sophisticated review on that topic). The use of multiconfigurational ab initio methods such as complete active space self-consistent-field (CASSCF) (Roos et al., 1980), restricted active space self-consistent-field (RASSCF) (Olsen et al., 1988; Malmqvist et al., 1990), generalized active space self-consistent-field (GASSCF; Olsen et al., 1988; Fleig et al., 2001; Dongxia et al., 2011) as well as extension to perturbation theory such as CASPT2, RASPT2, GASPT2, N-electron valence state second-order perturbation theory (NEVPT2) or multireference Møller-Plesset second-order perturbation theory (MRMPT2) is therefore required. Since those methods are extraordinarily expensive and non-trivial in their application they are often used to benchmark the performance of DFT functionals in the hope to get at least a qualitatively correct picture using a simpler and cheaper DFT-based method. Venturinelli Jannuzzi et al. (2016) compared the excited state energetics of manganese porphyrins employing several GGA functionals with and without the addition of direct Hartree-Fock (HF)-exchange against multiconfigurational methods such as CASPT2 and RASPT2. As expected they find that GGAs overstabilize low-spin states while the inclusion of HF exchange stabilizes high-spin states with respect to low-spin states. Thereby the amount of HF exchange determines the degree of stabilization. On the other hand the radical character determined by Mulliken population analysis was shown to be exaggerated if more than 15% HF exchange was employed. Those opposing trends make the choice of the functional and its HF exchange a crucial parameter. Ashley and Baik (2016) studied similar Mn^V^ = O complexes employing CASSCF and CASPT2 as well as B3LYP and variants thereof with different HF contributions, where they find a similar dependency of the radical character. Further they investigated whether the radical character influences the electronic barrier for the O−O bond formation either by a WNA or a RC. The barriers were found for both mechanisms to depend on the radical character, but the influence diminished after a certain threshold was reached. Those findings are in good agreement with CASSCF calculations which suggested only modest amount of oxyl radical. Those opposing trends make the choice of the functional and its HF exchange a crucial parameter. Ashley and Baik (2016) studied similar Mn^V^ = O complexes employing CASSCF and CASPT2 as well as B3LYP and variants thereof with different HF contributions, where they find a similar dependency of the radical character. Further they investigated whether the radical character influences the electronic barrier for the O−O bond formation either by a WNA or a RC. The barriers were found for both mechanisms to depend on the radical character, but the influence diminished after a certain threshold was reached. Those findings are in good agreement with CASSCF calculations which suggested only modest amount of oxyl radical.

The quality of multiconfigurational calculations strongly depends on the choice of an appropriate and sufficiently large active space, however the extraordinary high computational cost often becomes a limiting factor. An attempt to make large active spaces computational feasible is the density matrix renormalization group (DMRG) methodology (for fundamentals and recent developments, see e.g., Chan and Sharma, 2011; Olivares-Amaya et al., 2015; Guo et al., 2016; Ma et al., 2017; Freitag et al., 2017; Nakatani and Guo, 2017), which was for example employed by Kurashige et al. (2014) to a Fe^V^ = O dimer. They further added dynamic correlation effects by DMRG-CASPT2 and DMRG-multireference configuration interaction (MRCI) calculations. The largest active space employed in their study (36 electrons, 32 orbitals) resulted in a similar activation barrier for the O−O bond formation like DFT when using B3LYP and Grimme's D3 dispersion correction. However, significant differences between the multireference methods and DFT where found when comparing the stabilization of the product state, i.e., Fe^IV^−OO−Fe^IV^. Besides the CASPT2 formalism, NEVPT2 has experienced increased attention in the recent past. However, there are only few benchmark studies available. Pierloot et al. (2017) attempted to resolve this problem by benchmarking the performance of NEVPT2 in terms of the spin state energetics of 3d transition metal ions and complexes. They come to the rather disappointing conclusion that NEVPT2 is not a systematical improvement over CASPT2. However, those conclusions might be strongly dependent on the systems and the observables used. For example, Schapiro et al. (2013) reported NEVPT2 and CASPT2 to perform very similar in terms of vertical excitations energies for a set of organic molecules. A high-level alternative to DFT and other traditional quantum chemistry methods is the quantum Monte Carlo (QMC) approach. Chu et al. (2016) successfully applied QMC to calculated free energy profiles for water oxidation catalyzed by a Co^III^-aqua ion model system, where they find a fair agreement with CCSD(T) calculations. Further, the results qualitatively recovered free energy profiles obtained with the help of NEVPT2. The main reason not to use multireference methods is the unfortunate scaling behavior of most wavefunction based methods. Nevertheless, the development of multiconfigurational pair-DFT (Li Manni et al., 2014; Ghosh et al., 2015), heat bath configuration interaction (Holmes et al., 2016; Sharma et al., 2017), CAS-QMC (Li Manni et al., 2016), as well as the application of domain-based local pair natural orbital methods such as DLPNO-CCSD(T) (Sparta and Neese, 2014; Liakos et al., 2015; Saitow et al., 2017) highlight the fact that those methods and methodologies are subject to active research - an in depth overview of which is beyond the scope of this review.

### 3.4. Reduction potentials and other properties

Validation of the proposed mechanisms is usually quite challenging since the calculated intermediates often cannot be isolated nor characterized. Nevertheless, experimental reduction potentials and *pK*_*A*_ values are regularly available for one or more steps of the catalytic cycle and thereby might serve as a direct link to the experiment. Even though numerous protocols exist (for a recent review see Ho and Coote, 2009; Marenich et al., 2014), accurate calculation of the latter is still not a trivial task. Water oxidation normally takes place in the condensed phase, hence the choice of the solvation model is again of great importance.

The standard free energy of the electron affinity (EA, M^*n*+^ + e^−^ → M^(*n*−1)+^) in solution (sol), $\Delta G_{sol}^{{}^{\circ}EA}$, is related to the standard reduction potential *E*° by the Faraday constant (F),

$$\Delta G_{sol}^{{}^{\circ}EA}=-FE^{{}^{\circ}}$$

It is composed of the free energy of the EA in the gas phase (gas), $\Delta G_{gas}^{EA}$, and the associated solvation free energy Δ*G*_*solv*_. $\Delta G_{gas}^{EA}$ can be rewritten according to the definition of the *Gibbs* Free energy as the difference of the enthalpy $\Delta H_{gas}^{EA}$ and the Temperature multiplied by entropic contributions Δ*S*_*gas*_. The latter can be calculated by quantum chemical programs as the sum of difference in electronic energy ($\Delta E_{gas}^{EA}$), the difference in zero-point-energy (ΔZPE) and the difference of the thermal correction term Δ*H*^*T*^. The last term is usually obtained by applying the rigid-rotator approximation within a harmonic potential (Baik and Friesner, 2002):

$$\begin{array}{l} \Delta G_{sol}^{EA}=\Delta G_{gas}^{EA}+\Delta G_{solv}\\ \Delta G_{gas}^{EA}=\Delta H_{gas}^{EA}-T\Delta S_{gas}\\ \Delta H_{gas}^{EA}=\Delta E_{gas}^{EA}+\Delta ZPE+\Delta H^{T} \end{array}$$

The possibility to calculate $\Delta G_{sol}^{EA}$ from $\Delta G_{gas}^{EA}$ and Δ*G*_*solv*_ is often referred to as *Born-Haber* or thermochemical cycle, and is best known in the context of implicit solvation models (Wang et al., 2010). In order to calculate the change in free energy of an ET ($\Delta G_{gas}^{EA}$) or of the proton affinity (PA) in case of a PT ($\Delta G_{gas}^{PA}$), one needs to define the free energy of an electron and a proton respectively. There are common values which have been used on a regular basis in literature. However, to take full advantage of error cancellation sometimes an isodesmic scheme is used, where the electron in case of an ET respectively the proton in case of a PT is transferred between two species and never exists as a sole entity (see Keith et al., 2013). A disadvantage of those thermochemical cycles is that they do not capture structural rearrangements of the molecule due to solvation, if those play an important role it might be advisable to directly calculate $\Delta G_{sol}^{EA}$ without a thermochemical cycle (Ho, 2015). For certain molecules, in particular aqua-ions, it turned out that an explicit first solvation shell is necessary in order to obtain reduction potentials which are comparable to the experimental values. Also here, explicit solvation suffers from the sampling problem discussed earlier, therefore alternative solvation models were developed, such as COSMO-RS, which was found to be a significant improvement compared to the standard implicit solvation models such as COSMO or PCM (Rulíšek, 2013). Besides the directed bonding, implicit solvation models also struggle to deal with highly charged species, which however are an essential part of most catalytic water oxidation cycles. Bím et al. (2016) came up with the so called variable-temperature H-Atom addition/abstraction (VT-HAA) protocol where the calculation of highly charged species is avoided by employing thermochemical cycles. For a set of 15 charged TM complexes, it was shown that the VT-HAA protocol significantly improves the agreement of predicted reduction potentials with experimental data. Beyond that there are more sophisticated protocols that apply AIMD to explicitly solvated systems in order to determine *E*_*red*_ and *pK*_*A*_ values (Cheng et al., 2009; Sulpizi and Sprik, 2010). In the context of water oxidation the dehydrogenation free energy Δ*G*(PCET) is of particular interest, which is also accessible by a related protocol, as has been shown by Hodel and Luber (2017).

For a computational chemist, a reduction is rather straight forward by increasing the number of electrons in the studied system. However, an experimental chemist is especially interested in oxidation states of certain atoms, e.g., the transition metals. This is problematic since the concept of oxidation states lacks a rigorous theoretical foundation at a quantum mechanical level. There are many electronic-structure based methods to determine the oxidation state by either partitioning the total charge density among atoms and ions such as Bader, Voroni charges or projection techniques such as Mulliken charges or Löwdin charges (Sit et al., 2011). Each of those methods has its advantages and flaws which will not be discussed here. For transition metal complexes, where an ionic metal center is surrounded by neutral or ionic ligands such methods often reach their limitation. There have been several concepts suggested based on localization procedures, none of which has yet become the standard tool. However, they are often able to give a picture which is closely related to concept of oxidation states (Aullón and Alvarez, 2009; Thom et al., 2009; Sit et al., 2011; Reeves and Kanai, 2014; Vidossich and Lledos, 2014).

On a side note, we expect that relativistic effects should have a negligible contribution toward all the properties discussed in this section, since they become usually only relevant for elements with an atomic number larger than 50 (Bond, 2000). However, for certain applications, in particular in the context of spectroscopy, inclusion of relativistic effects might still be beneficial (Kumagai et al., 2009).

In the following section, recent developments in the field of homogeneous transition metal catalysts for water oxidation will be discussed. Thereby we will focus on cobalt-based WOCs, which have been shown to be e.g., stable mimics for nature's OEC. Further, homogeneous model systems might help to elucidate the water oxidation reaction mechanism of the well-known heterogeneous CoO_*x*_. For other well studied transition metal WOCs we refer to recent reviews (e.g., Kärkäs et al., 2014; Sala et al., 2014; Blakemore et al., 2015; Thomsen et al., 2015; Kondo and Masaoka, 2016; Najafpour et al., 2016).

## 4. Cobalt-based water oxidation catalysts

**Figure 5:**
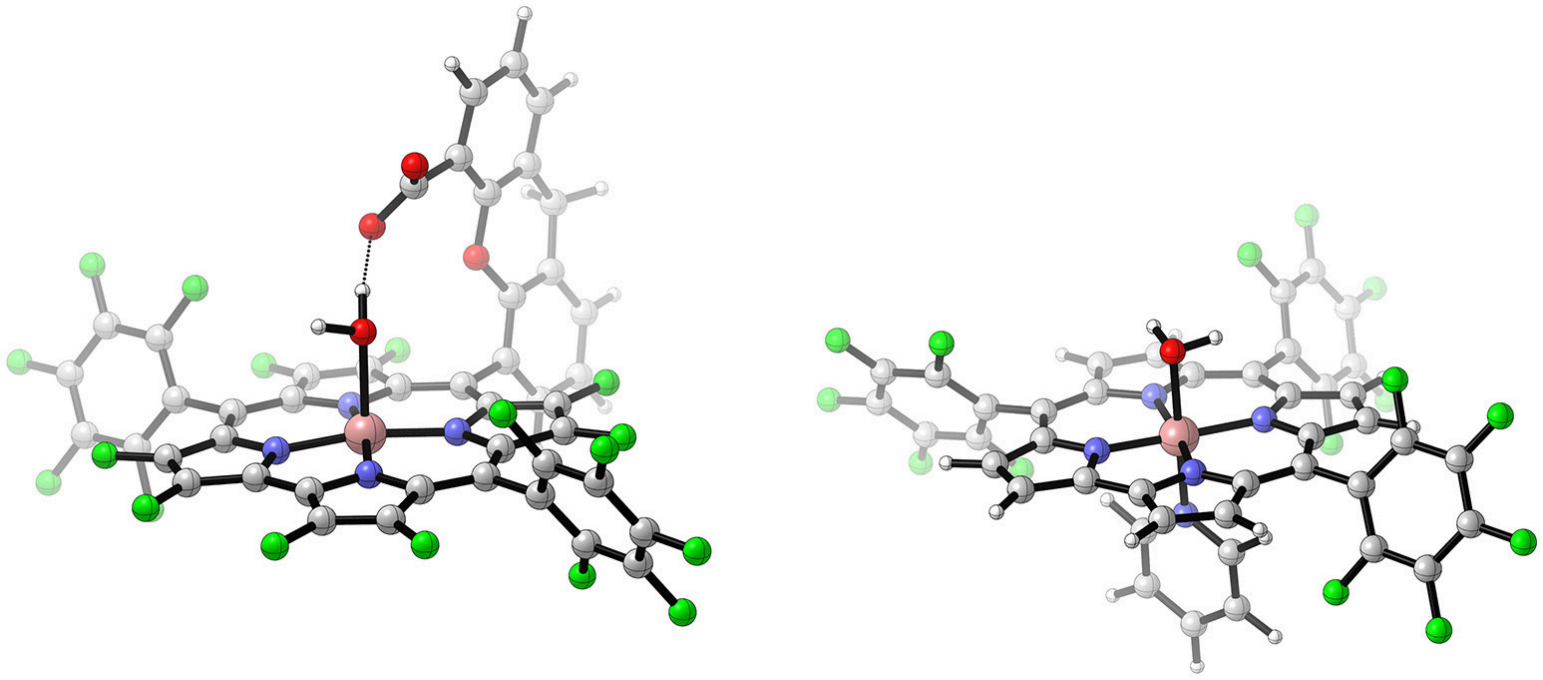
Schematic illustration of a Hangman-Corrole studied by Lai et al. (2012) **(left)**, and an ordinary corrole complex **(right)** the mechanism of which was modeled by Lei et al. (2014). The following color code applies for all illustrations: *white*-hydrogen, *gray*-carbon, *dark blue*-nitrogen, *red*-oxygen, *green*-fluorine, *light pink*-cobalt. Reproduced from geometrical data published by Lai et al. (2012) **(left)** and Lei et al. (2014) **(right)**.

Computational studies on the water oxidation mechanism of Co-based WOCs are rather scarce. Nevertheless, some WOCs were studied with great care, among them are the so-called Hangman-Corroles (Dogutan et al., 2011). Those molecules feature a β-octafluoro corrole (cor) with a linker bearing a carboxyl acid. The latter is placed above the face of the macro-cycle and can act as a proton acceptor or proton donor; this distinctive structural feature is known as Hangman construct (see Figure 5) (Rosenthal and Nocera, 2007).

Based on calculations of reduction potentials, free energies of deprotonations, PCETs, as well as transition states for the O−O bond formation by a WNA and the displacement of molecular oxygen by a solvent molecule, the following reaction mechanism was proposed for the Hangman-Corroles: Starting from a Co^III^−OH_2_ state, two consecutive PCETs lead to a reactive state, where a WNA takes place, the carboxylate of the Hangman construct thereby acting as an intra-molecular base and facilitating the O−O bond formation. After an additional PCET, either deprotonation of the carboxylic acid followed by the substitution of molecular oxygen by a solvent molecule or a fourth PCET takes place after which the displacement of the superoxide by a water molecule occurs. Applying the nomenclature established earlier, the mechanism can be described as: PCET-PCET-[(i-PT)-WNA]-PCET-PT-O2DI-AQAS-ET or PCET-PCET-[(i-PT)-WNA]-PCET-PCET-[O2DI-AQAS]. A key factor determining the likelihood for a WNA is the electrophilicity of the oxo / oxyl ligand, which in turn is governed by the oxidation state of the metal center. Here, the WNA takes place at a formal Co^V^ center. However, calculations by Ertem and Cramer (2012) indicate radical character on the oxo ligand and on the corrole frame work. Those two non-innocent ligands are formally oxidized instead of the metal center. This results in an electronic structure which cannot be described by standard DFT anymore, therefore the broken-symmetry approach was employed to obtain the energies of the anti ferromagnetically-coupled spin system.

The influence of different transition metals on the activation barrier was studied by Lai et al. (2012). They compared activation barriers calculated for formal [M^V^(cor−COO)(O)]^−^ and [M^IV^(cor−COO)(O)]^2−^ intermediates (M = Co, Mn, Fe, Ru, and Ir). While the [M^V^(cor−COO)(O)]^−^ state undergoes a WNA that is equivalent to the equivalent to the one proposed by Ertem and Cramer (2012), [M^IV^(cor−COO)(O)]^2−^ represents an intermediate from catalytic cycle (lower formal oxidation state before the O−O bond formation) which supposedly starts with the steps PCET-PT-WNA. Since the transition metal center and its oxidation state guide the water oxidation mechanism, a clear preference for the higher oxidation state is expected. It is also important to note that the formal oxidation states not necessarily represent the observed oxidation state, since the ligands are non-innocent (Ertem and Cramer, 2012). Lai et al. (2012) reported barriers ranging from 3.6 kcal/mol for Co^V^ up to 58.3 kcal/mol for Mn^IV^ for the O−O bond formation. As expected, in general a higher oxidation state favors a WNA attack, which can easily be rationalized by the stronger electrophilic character of the catalyst. Further they argue that in the series of the 3d transition metals the ability for two electron reduction governs the reactivity. The latter is defined as electronic energy difference for the [(i-PT)+WNA] step, which can be thought of as a PT, followed by two single ETs from OH^−^ to the catalyst, and finally an attack of the complex to a OH^+^(Lai et al., 2012).

A structural analog of the Hangman-Corroles without an intramolecular base was investigated by Lei et al. (2014) (see Figure 5). The main structural difference is the assumed octahedral coordination of the metal center compared to a quadratic pyramidal coordination in case of the Hangman-Corroles. Here, two pyridines occupy the coordination sites above and below the corrole plane. The assumed catalytic ground state [Co^III^(cor)(OH_2_)(py)] is reached after the substitution of one pyridine by a water molecule from the solvent. The dissociation of the pyridine thus becomes a gateway step toward the catalytic cycle, which is backed-up by experimental studies. The proposed water oxidation mechanism PCET-PCET-[WNA-PT]-PCET-ET-[O2DI-AQAS] is similar to the one proposed by Ertem and Cramer (2012). The barrier for the O−O bond formation (29.9 kcal/mol) could be reduced by more than 10 kcal/mol if an intermolecular base “OAc^−^,” was included into the transition state model. The reactive intermediate is again best described as a [Co^III^(Cor^•+^)(O^•+^)(py)] species rather than a [Co^V^(cor)(O)(py)]. Recently, a series of Co-corroles was reinvestigated by Ganguly et al. (2017) employing BS-DFT calculations. They find that the presumed Co^III^(cor) state has significant contributions of an anti-ferromagnetically coupled Co^II^(cor^•^) state, highlighting the fact that corroles are non-innocent. If those findings are also true for the WOCs discussed earlier, and whether the latter would have any influence on the reaction mechanism is still under discussion.

**Figure 6:**
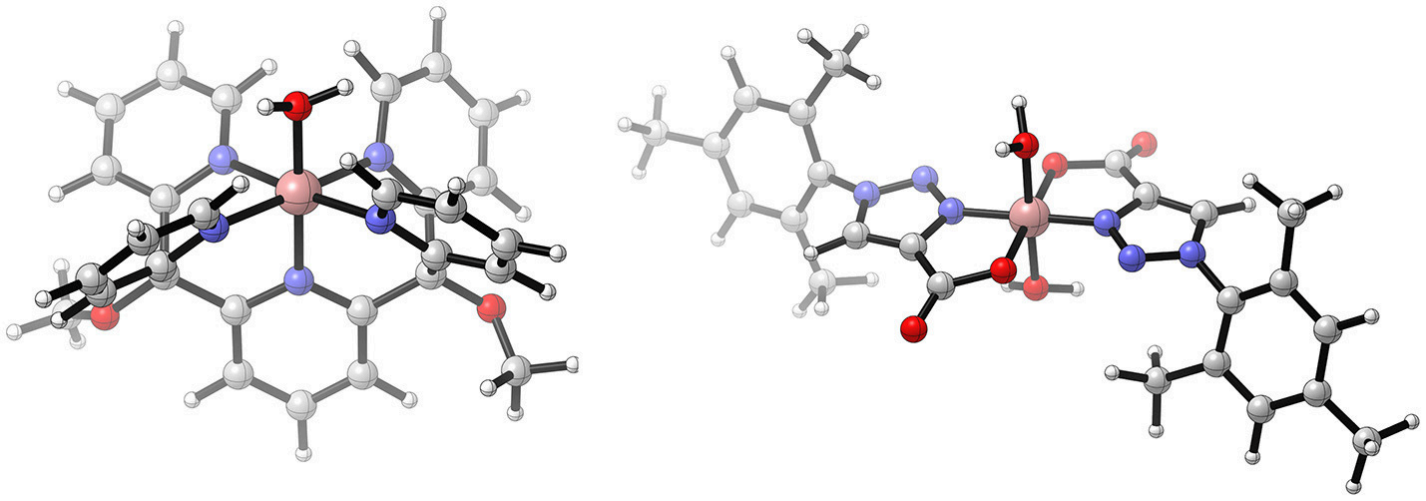
Schematic illustration of [Co^II^(Py5)(OH_2_)]^2+^ investigated by Crandell et al. (2015) **(left)**, and [Co^II^(TCA)_2_(OH_2_)_2_] studied by Younus et al. (2017). Reproduced from geometrical data published by Crandell et al. (2015) **(left)** and Younus et al. (2017) **(right)**.

**Figure 7:**
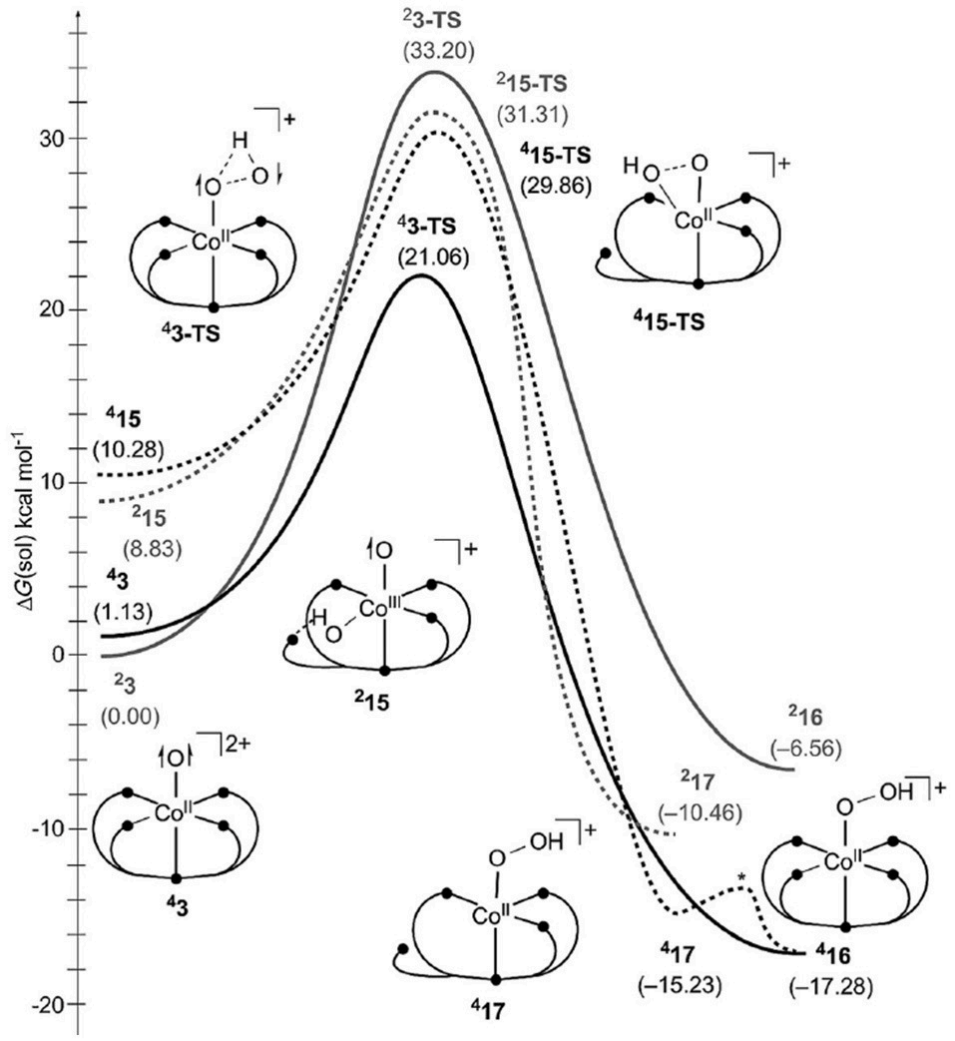
Free energy profile of the O−O bond formation catalyzed by [Co^II^(Py_5_)(OH_2_)]^2+^. Note the associative mechanism where the nucleophile first coordinates to the cobalt center. Crandell et al. (2015), Copyright Wiley-VCH Verlag GmbH & Co. KGaA. Reproduced with permission.

A common structural feature of most WOCs is the coordination by polydentate nitrogen bearing ligands such as corroles, porphyrines, or pyridine derivatives. Another catalyst with such a pyridine ligand scaffold is [Co^II^(Py5)(OH_2_)]^2+^ [Py5 = 2,6-(bis(bis-2-pyridyl)-methoxymethane)pyridine] (see Figure 6; Wasylenko et al., 2011). In contrast to the WOCs described above, it is the first time that the catalytic ground state contains Co^II^ instead of Co^III^. This has implications on the reaction mechanism: first, the ligand sphere of Co^II^ complexes is prone to ligand exchange, secondly high oxidation states such as Co^V^ can be avoided. On the other side, the lower oxidation state in principle reduces the electrophilicity of the reactive Co^III^−O^•^ species which in turn makes a WNA mechanism less likely. Nevertheless, the catalytic cycle proposed by Crandell et al. (2015) follows the standard pattern established before. After two consecutive PCETs, a nucleophilic attack of a hydroxide on the oxyl-species takes place. A subsequent two electron oxidation and deprotonation results in the release of molecular oxygen, and upon coordination of a water molecule the catalytic ground state is recovered (PCET-PCET-WNA(OH)-[PCET+ET]-[O2DI-AQAS]). In their study, Crandell et al. (2015) also investigate whether the WNA is an intra- or intermolecular process, since in principle a hydroxide molecule could coordinate to the metal center prior to the nucleophilic attack onto the oxyl-species, a possibility which has often been overlooked in similar studies. Because the metal center is already coordinatively saturated at the reactive intermediate (Co^III^−O^•^), and ligand exchange reactions are expected to be unlikely at highly oxidized cobalt centers, decoordination of a pyridine at an earlier stage of the catalytic cycle is required. The barrier for the substitution of an equatorial pyridine by a hydroxide in the catalytic ground state was found to be 18.7 kcal/mol, which might be overcome under experimental conditions. The barrier for the WNA itself was found to be smaller for an intramolecular (21.1 kcal/mol) than for an intermolecular mechanism (29.9 kcal/mol), but if the relative stability of the oxyl-species is taken into account (8.8 kcal/mol, in favor of the η^5^-Py5 species) both mechanisms become energetically equivalent. In this case, the intermolecular mechanism is expected to dominate because of the energy penalty associated with the coordination of hydroxide (structures where a pyridine ligand is dissociated are higher in free energy - see Figure 7).

Recently, Younus et al. (2017) reported a mononuclear Co-catalyst with a completely different ligand frame work (see Figure 6). The latter is composed of bidentate triazol-carboxylates that coordinate with one oxygen of the carboxylate as well as one nitrogen atom of the triazol to the metal center. The final complex is composed of two of those ligands in a trans configuration [Co^II^(TCA)_2_(OH_2_)_2_] (TCA = 1-mesityl-1,2,3-1H-triazole-4-carboxylate). Based on experimental data the following reaction mechanism was proposed: PCET-PCET-[WNA-PCET]-PCET-[O2DI-AQAS]. The two alternative pathways, i.e., RC and a bifunctional mechanism, were also considered. However, a RC mechanism could be excluded by experiments, and the bifunctional mechanism was ruled out since there is no suitable hydrogen acceptor in close proximity to facilitate a concerted mechanism.

**Figure 8:**
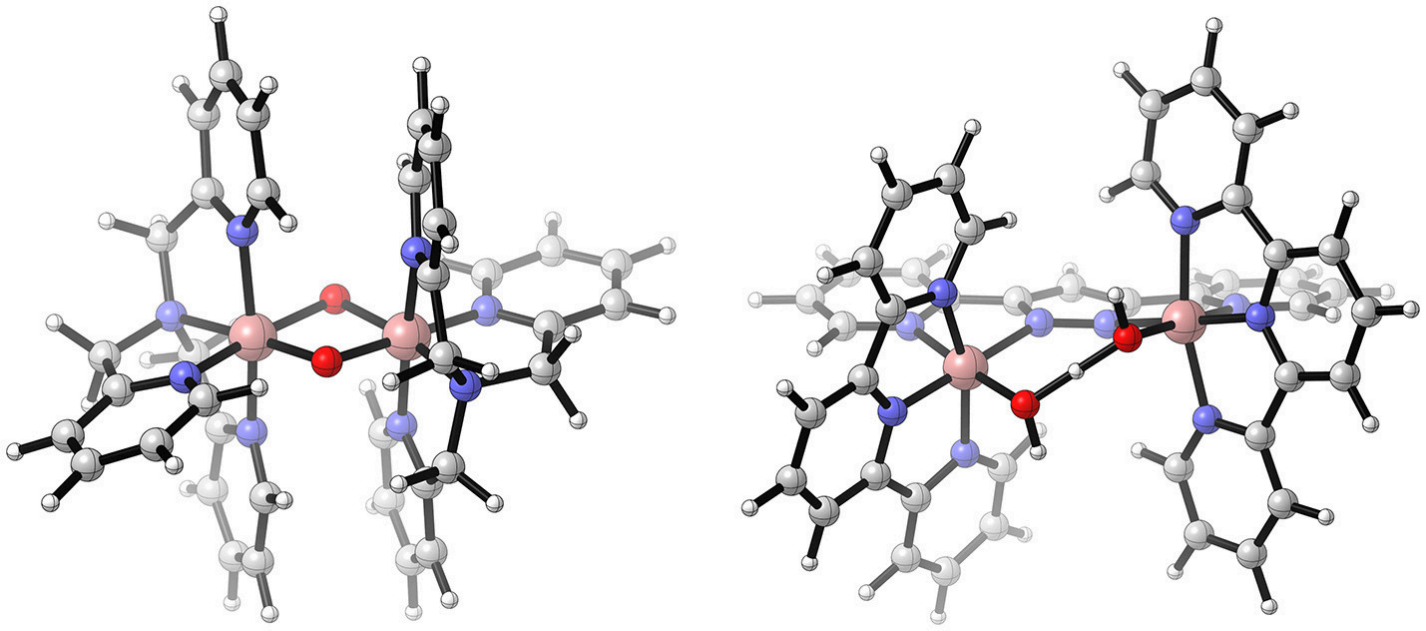
Schematic illustration of [${\text{Co}}_{2}^{\text{III}}$(TPA)_2_(μ−O)_2_]^2+^ as studied by Ishizuka et al. (2016) **(left)**, and (Co^III^(trpy))_2_(μ−bpp)(OH_2_)(OH))^4+^ by Gimbert-Suriãch et al. (2016) **(right)**. Reproduced from geometrical data published by Ishizuka et al. (2016) **(left)** and Gimbert-Suriãch et al. (2016) **(right)**.

Moving on from mononuclear Co-based WOCs to dinuclear catalysts, only a few examples of mechanistic studies can be found in the literature. An example is [${\text{Co}}_{2}^{\text{III}}$(TPA)_2_(OH)_2_]^4+^ (TPA = tris(2-pyridylmethyl)amine) by Ishizuka et al. (2016) (see Figure 8). The main structural feature of those complexes are the two bridging hydroxyl ligands that are not only crucial for the formation of the dimeric structure, they are also the substrate for the O−O bond formation. The proposed mechanism can be abbreviated as PT-[ET-PCET]-(i-RC(μ − *O*))-[AQAS-PT]-ET-ET-O2DI-AQAS-PT-PT, where the first AQAS-PT refers to an insertion of a H_2_O molecule in between the metal centers forming a new hydroxyl bridge. Employing DFT calculations (Ishizuka et al., 2016) characterized the spin state of the catalytic intermediate which undergoes the O−O bond formation, [${\text{Co}}_{2}^{\text{III}}$(TPA)_2_(O^•^)_2_]^4+^. For the latter, a triplet state was found to be most stable, suggesting the formation of two μ-oxyl species. Formation of the O−O bond leads to a formal superoxide which is coordinated in a side-on (η^2^) fashion to each of the two metal centers. Upon coordination of a solvent molecule the coordination mode changes to endo-on (η^1^), and subsequent oxidation leads to the release of molecular oxygen.

A dinuclear WOC [(Co^III^(trpy))_2_(μ−bpp)(μ−OO)]^3+^ (trpy = 2,2′;6′:2″-terpyridine; bpp^−^ = bis(2-pyridyl)-3,5-pyrazolate), which was found to be also a hydrogen reduction catalyst, was reported by Fukuzumi et al. (2012), Rigsby et al. (2012), and Gimbert-Suriãch et al. (2016). The mechanism governing water reduction was experimentally elucidated by Mandal et al. (2013) and Di Giovanni et al. (2016). Unlike most dinuclear WOCs, it does not possess any bridging oxo-ligands, the pyrazol subunit of the bbp ligand takes over this role. The other coordination sites of the octahedrally coordinated metal centers are occupied by trpy ligands as well as solvent molecules which are the substrate for the water oxidation (see Figure 8). The proposed mechanism for water oxidation is initiated by a formal [(Co^III^(trpy)(OH_2_))(μ−bpp)(Co^III^(trpy)(OH))]^4+^ species, the latter undergoes two PCETs, a deprotonation, an O−O bond formation by RC, followed by regeneration of the initial state by H_2_O insertions and further oxidations. In summary: PCET-PCET-[PT-(i-RC)]-ET-AQAS-PCET-[O2DI-AQAS] (at pH = 2). A remarkable feature of the proposed catalytic cycle is the fact that even though the resting state is composed of two Co^III^ centers, none of them is expected to be oxidized to a formal oxidation state higher than Co^IV^ (formally Co^III^-oxyl). The mechanism is backed up by exhaustive calculations of dehydrogenation free energies (PCETs), redox-potentials, and pKa values for different conditions (pH-values), as well as activation energies for the O−O formation, the change of the coordination mode of O_2_ from η^2^ to η^1^, and the O_2_ release.

**Figure 9:**
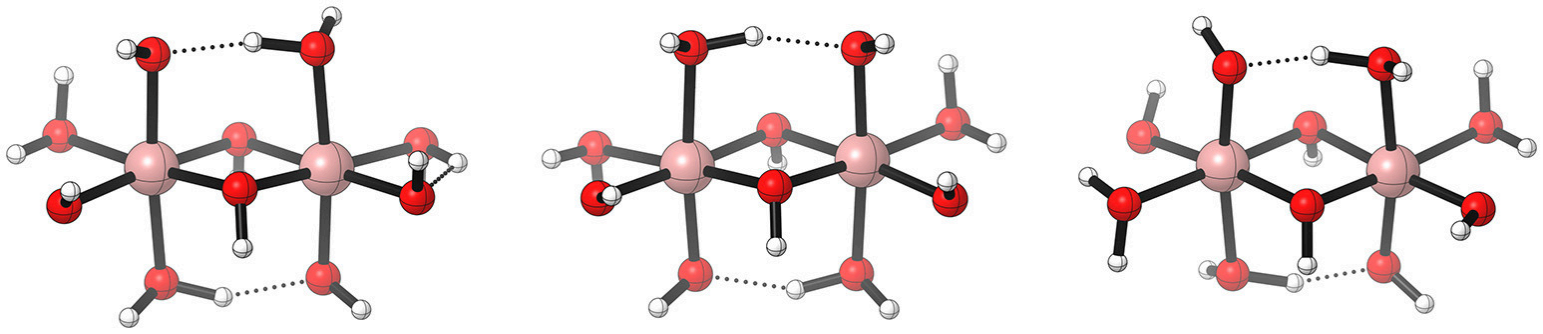
Schematic illustration of three [(Co^III^(OH_2_)_2_(OH)_2_)_2_(μ−OH)_2_] isomers. Electronic energy difference given relative to the left structure, 0.0 eV **(left)**, 0.12 eV **(middle)**, 0.21 eV **(right)**. Reproduced from geometrical data published by Fernando and Aikens (2015).

Fernando and Aikens (2015) studied possible reaction mechanisms for a dinuclear model system of cobalt oxide [(Co^III^(OH_2_)_2_(OH)_2_)_2_(μ−OH)_2_], which does not contain any organic ligands. Obviously there are many protonation isomers, therefore the complexity quickly increases along the possible reaction path. For example, there exist 14 possibilities for the first PCET, then for each of the isomers obtained after the first PCET, there are 13 possibilities for the second PCET, and so on. The stability of those isomers is largely determined by their intramolecular hydrogen bonding network which may be too favored due to the use of a solvent continuum model (see Figure 9). With the given structures there are plenty of possible pathways for the O−O formation. The ones described in the study can be grouped into intra- and intermolecular WNAs, where the nucleophile is either a water molecule, a μ–OH or a geminal OH. Fernando and Aikens (2015) report the WNA pathway (PCET-PCET-PCET-WNA-PCET-O2DI-AQAS) to be thermodynamically favorable over the two possible RC mechanisms, but no barriers were calculated which support this argument.

The active species for all three possible nucleophiles is reported to be a Co^V^−O^•^ (formally Co^VI^ = O) obtained after three consecutive PCETs, all of which have to happen at the same metal center. While the authors do not attempt to assign formal oxidation states to the individual metal centers, the nomenclature of the oxyl as well as the reported spin multiplicity clearly suggest a single-site mechanism. In this light, the question arises why the second metal center is even necessary, a question which was not discussed in the study. So why is the dinuclear model system still important? It might be envisioned to be the smallest building block of Co_x_O_3_, which is known to be a potent heterogeneous WOC, even more since Kanan and Nocera (2008) reported their famous CoP_i_ catalyst. The next larger subunit—trinuclear complexes complexes such as [Co_3_(O)(OH)_2_(OAc)_3_(py)_5_]^2+^—were found to decompose under catalytic conditions and therefore were not studied in great depth (Li and Siegbahn, 2013; Smith et al., 2014).

**Figure 10:**
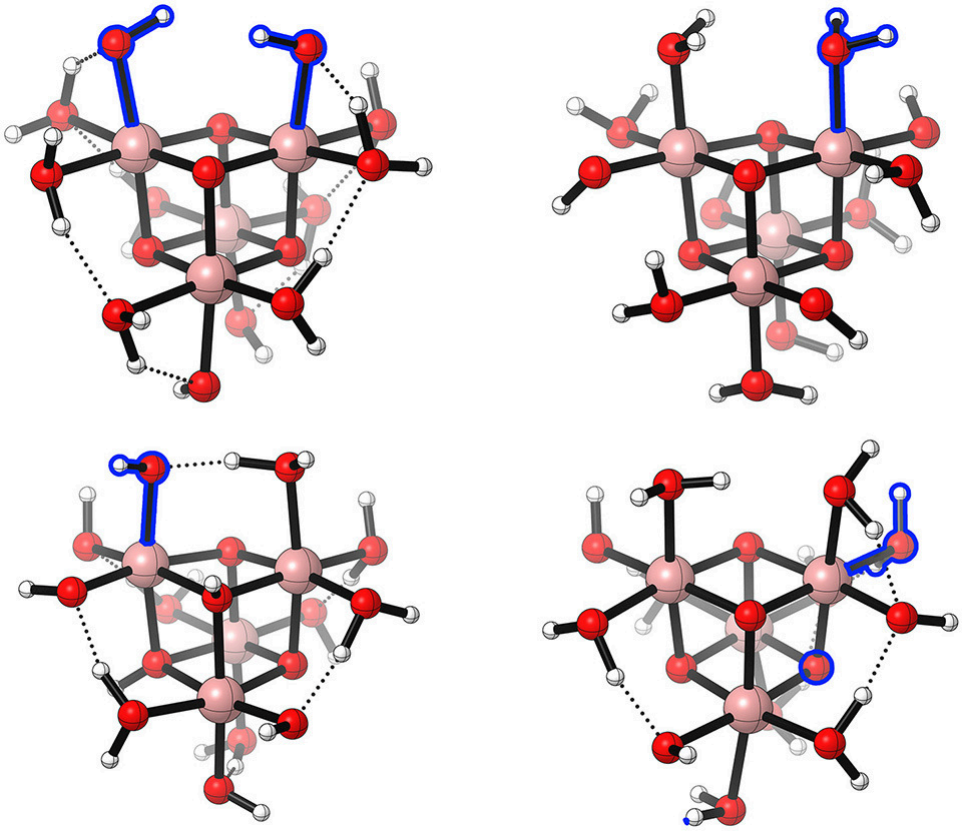
Schematic illustration of the [(Co^III^(OH_2_)_2_(OH)(μ^3^-O))_4_] model system as used by Wang and Van Voorhis (2011) **(top, left)**, Kwapien et al. (2013) **(top, right)**, Li and Siegbahn (2013) **(bottom, left)**, and Fernando and Aikens (2015) **(bottom, right)**. If indicated in the original paper, hydrogen bonds are shown. Further, the “active” ligands, i.e., the ones which undergo the O−O bond formation, are highlighted in blue. Reproduced from geometrical data published by Wang and Van Voorhis (2011) **(top, left)**, Kwapien et al. (2013) **(top, right)**, Li and Siegbahn (2013) **(bottom, left)**, and Fernando and Aikens (2015) **(bottom, right)**.

Catalytically active and stable polynuclear WOCs with four centers exhibit a cuboidal structure (see Figure 10). Like for the dinuclear species, there is a large number of possible protonation isomers. Fernando and Aikens (2015) selected the thermodynamically most stable ones for further investigations. They again considered different pathways for the O−O bond formation - a WNA, an i-WNA, i-RC(geminal), and i-RC. Here it is worth to mention that the bridging oxygens are not protonated (μ–O) in their model (see Figure 10). From a thermodynamical point of view, radical coupling between an oxyl and a bridging oxygen turned out to be favorable (PCET-PCET-(i-RC(μ–O))-AQAS-(AQAS-PCET)-O2DI-PCET). A different protonation isomer, where the two hydroxyl ligands of Co are on the same site, was found to be energetically unfavorable. Nevertheless, it might be a more realistic model for Co_x_O_3_ surface. For those models, an additional pathway arises, namely i-RC between two oxo species. All discussed pathways share a common starting sequence of reactions – two consecutive PCETs, leading to Co^IV^−O^•^ or Co^V^=O. The authors use a slightly misleading nomenclature (Co^V^−O^•^ radical), which would imply a formal oxidation state of Co^VI^ for the Co-oxo species. However, such a high oxidation state can only be reached after three oxidation steps (see dimer). The electrophilicity of Co^V^−O^•^ and Co^IV^−O^•^ is expected to be significantly different, which might explain the preference for a RC mechanism over a WNA. Even though this model system oversimplifies aspects of a real system such as solute-solvent interactions, it is one of the few studies which shows the complexity arising from different protonation sites. For real catalysts, those considerations might not be as important as for the model system since the number of acidic / basic sites is often limited. Nevertheless, there might be the possibility of ligand decoordination or substitution which might result in a large number of protonation isomers. To overcome the flaws of implicit solvation models, in particular the treatment of hydrogen bonding between the solute and the solvent, Wang and Van Voorhis (2011) placed the same model system as used by Fernando and Aikens (2015) in a box of 216 water molecules and treated the whole system on a QM/MM level of theory. They chose a protonation state where two hydroxyl ligands are at the same face of the cubane (see Figure 10). Those can then undergo i-RC. They also calculated activation barriers to discriminate certain pathways, among them was a WNA, an i-WNA(OH), as well as i-RC between two bridging oxo-ligands. Energetically all of those were found to be inferior to the standard i-RC between two Co^III^−O^•^ located on the same face of the cubane. The reaction path might be summarized as PCET-PCET-(i-RC)-[AQAS-PCET-PCET]-[O2DI-AQAS]. Like Fernando and Aikens (2015), they find that only two PCETs are necessary to arrive at catalytically active oxyl species (Co^III^−O^•^). It is important to note that the spin state of the intermediate prior to the O−O bond formation is an open-shell singlet (i.e., anti ferromagnetic-coupling between the metal centers). However, the ferromagnetic triplet state was found to be only 1.6 kcal/mol higher in electronic energy. An interesting observation is also that the activation energy for an i-WNA by a hydroxyl species was found to be much higher than the one for an i-RC mechanism. This might raise the question how large the barriers for an i-RC are in case of the dinuclear model complex discussed by Fernando and Aikens (2015). In their conclusion Wang and Van Voorhis (2011) challenge one of the key assumptions most experimentalists and theoreticians make, by proposing that either the addition of a water molecule or an intramolecular PT might be rate-limiting rather than the O−O formation. While based on experimental conditions (e.g., the presence of strong oxidants or bases) it often can be justified that ET and intermolecular PT steps are irreversible and not rate-limiting, this is not necessarily true for chemical steps (e.g., O−O bond formation, O_2_ release, or intramolecular PTs).

Kwapien et al. (2013) used the same model system [(Co^III^(OH_2_)_2_(OH)(μ^3^-O))_4_] (see Figure 10) and the smallest possible subunit of it – an aqua ion [Co^III^(OH_2_)_3_(OH)_3_] – to answer the question whether the choice of the exchange-correlation density functional has an effect on the structure of the intermediates and their reaction energies. In their study they assumed a classical single site mechanism (PCET-PCET-WNA-PCET-PCET-O2DI-AQAS) for both model systems. When comparing the hybrid functionals B3LYP and PBE0 with the GGA functional PBE, significant deviations were found in terms of the energetic ordering of different isomers, i.e., the thermodynamically favored product when the {H_2_O−Co−OOH} fragment undergoes a PCET (superoxo {H_2_O−Co−OO} or hydroperoxo fragment {HO−Co−OOH}). To further validate the density functionals, the electronic energy differences for the mononuclear Co-complex were compared with ones obtained from CCSD(T) calculations. A good agreement of both hybrid functionals, from a quantitative and qualitative point of view, with the CCSD(T) results was found. Such benchmark studies are important, in particular since the tetranuclear systems of interest are usually too complex for the application of a more accurate level of theory beyond DFT.

A different protonation state of the same model system as discussed above, where two bridging oxo ligands bear a proton in the resting state, was investigated by Li and Siegbahn (2013) (see Figure 10). The latter was found to be considerably lower in free energy than the model used by Fernando and Aikens (2015) and Wang and Van Voorhis (2011) (see Figure 10). The minimum energy reaction pathway proposed by Li and Siegbahn (2013) is similar to the already discussed one. Starting from the all Co^III^ resting state, two consecutive PCETs take place. Interestingly, the successive oxidation of a single cobalt center is favored over the oxidation of two equivalent centers. The resulting Co^V^=O then undergoes a WNA, followed by two PCETs and the release of molecular oxygen (PCET-PCET-[WNA-PCET]-PCET-[O2DI-AQAS]). Other reaction pathways were found to be energetically unfavorable because they contain either high energy intermediates or the barrier for the O−O formation was found to be too large. Even larger building blocks mimicking the heterogeneous Co_*x*_O_3_ catalyst were furthermore investigated by Mattioli et al. (2013) using AIMD and DFT+U, where the Coulomb interaction of localized electrons is treated with a Hubbard term (U).

**Figure 11:**
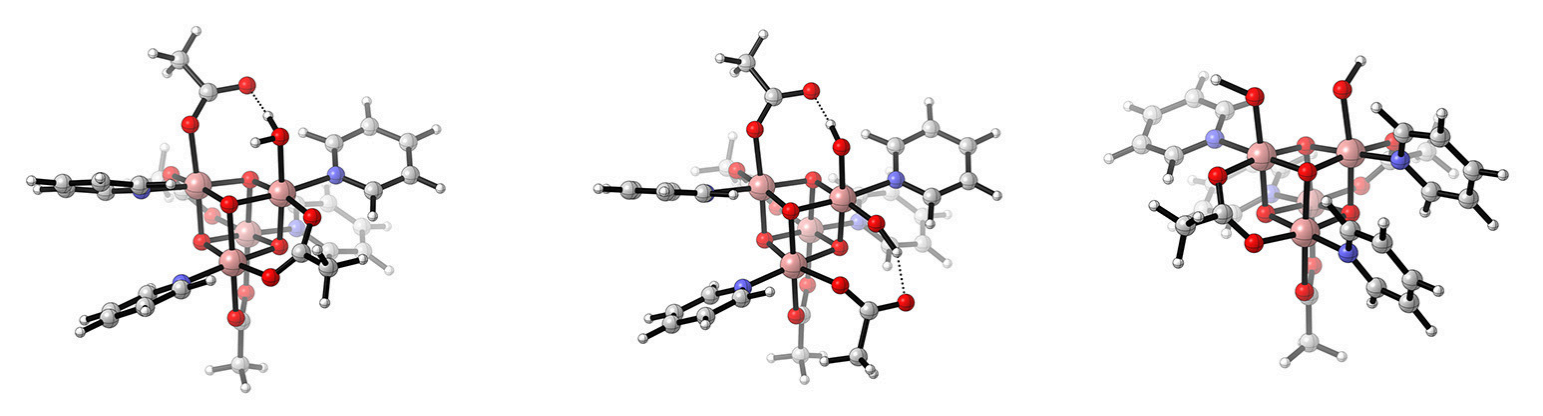
Schematic illustration of [${\text{Co}}_{4}^{\text{III}}$(μ^3^-O)_4_(μ^2^-OAc)_4_(py)_4_] where a single water molecule is associated (**left**, Li and Siegbahn (2013)), or either two hydroxyl ligands in a geminal- (**middle**, Smith et al. (2015)) or *cis*-configuration (**right**, Nguyen et al. (2017)). Reproduced from geometrical data published by Li and Siegbahn (2013) **(left)**, Smith et al. (2015) **(middle)**, and Nguyen et al. (2017) **(right)**.

A molecular catalyst featuring a cubodial core is [${\text{Co}}_{4}^{\text{III}}$(μ^3^-O)_4_(μ^2^-OAc)_4_(py)_4_], often referred to as *Dismukes-cubane* (McCool et al., 2011; La Ganga et al., 2012). Because of its structural similarity to their model system Li and Siegbahn (2013) applied the same protocol to this catalyst as well. It turned out that the first steps of the mechanism proposed for the model system also represent the minimum energy pathway for the *Dismukes-cubane*. The close proximity of an OAc^−^ to the active site Co^V^=O further allows it to act as an intramolecular base, which facilitates the WNA (see Figure 11). This system possesses a lower overall barrier than the model system, however like in the experiments a stronger oxidant with a reduction potential of 1.43 V instead of the theoretical 1.29 V was used.

Since the *Dismukes-cubane* does not feature an aqua or hydroxy ligand, which is a prerequisite for water oxidation, binding of a H_2_O or OH^−^ has to occur prior to entering the catalytic cycle. The latter was experimentally observed by Nguyen et al. (2015). They were also able to isolate a singly oxidized form of the catalyst, [${\text{Co}}_{3}^{\text{III}}$Co^IV^(μ^3^-O)_4_(μ^2^-OAc)_3_(OAc)(py)_4_]. The latter is able to evolve molecular oxygen upon addition of hydroxide. Since the reduction potential to reach a Co^V^ state is too large, they suspected [${\text{Co}}_{3}^{\text{III}}$Co^IV^(μ^3^-O)_4_(OH)(μ^2^-OAc)_3_(OAc)(py)_4_] to disproportionate. In principle the mechanism proposed by Nguyen et al. (2015) (ET-PT-ET-WNA(OH)-PCET-[ET-A2AS(OH)-O2DI]) is in agreement with earlier proposals for model systems by Li and Siegbahn (2013) and Kwapien et al. (2013), which both suggested a WNA after two consecutive oxidations. The same question, how an insufficient oxidant still could oxidize [${\text{Co}}_{3}^{\text{III}}$Co^IV^(μ^3^-O)_4_(OH)(μ^2^-OAc)_3_(OAc)(py)_4_] was also investigated by Smith et al. (2015). They argue that the association of a second hydroxyl ligand will lower the reduction potential by increasing the electron density in the cubane core and therefore reaching a Co^V^ state becomes feasible. The stability of several possible substitution patterns for the two hydroxyl ligands such as a *gem, cis*, and *trans* alignment were investigated. Computational results were not able to distinguish between the *cis*- and *gem*-isomers, since they were found to be isoenergetic. Yet, experiments suggested that no ligand dissociation occurs, a *cis* alignment can therefore be excluded which would require disassociation of an acetate ligand. The geminal alignment of the two hydroxyl ligands would potentially allow a WNA or an i-WNA(OH) after a subsequent PCET or an i-RC mechanism after two PCETs. However, they did not present any activation barriers for the O−O formation. Since the reduction potential was found to be dependent on the number of aqua, hydroxyl or oxo ligands, Nguyen et al. (2017) started a new attempt to elucidate the reaction mechanism by determining reduction potentials for related cubane structures using both experimental and theoretical tools. They identify [${\text{Co}}_{3}^{\text{III}}$Co^IV^(μ^3^-O)_4_(OH)_2_(μ^2^-OAc)_3_(py)_4_] as the thermodynamically most likely structure which disproportionates into the active species [${\text{Co}}_{3}^{\text{III}}$Co^V^(μ^3^-O)_4_(OH)(O)(μ^2^-OAc)_3_(py)_4_]. Adopting the earlier proposed mechanism to the new active species results in ET-PT-ET-[i-WNA(OH)])-PCET-[AQAS(2^*^OH^−^)-O2DI-ET], where the hydroxyl coordinated to a Co^III^ center nucleophilically attacks Co^V^−oxo (where AQAS(2^*^OH^−^) stands for the association of two hydroxide molecules).

Recently, Brodsky et al. (2017) challenged the single-site mechanisms discussed above by an experimental study where they were able to characterize the doubly oxidized species [${\text{Co}}_{2}^{\text{II}}$${\text{Co}}_{2}^{\text{IV}}$O_4_(μ^2^-OAc)_4_(py)]^2+^. Based on cyclic voltammetry measurements, X-ray absorption spectroscopy as well as BS-DFT calculations, they concluded that the species contains an anti ferromagnetically-coupled {${\text{Co}}_{2}^{\text{II}}$${\text{Co}}_{2}^{\text{IV}}$} core rather than a {${\text{Co}}_{3}^{\text{III}}$Co^V^} core. If those findings also hold for a species with some of the ligands replaced by aqua or hydroxyl ligands, they would in principle rule out a single-site mechanism and favor an i-RC as proposed by Wang and Van Voorhis (2011) for their model system.

Besides the Co^III^-based cubanes of McCool et al. (2011) there is a family of Co^II^ cubanes which has been investigated by Evangelisti et al. (2013, 2015), and Song et al. (2017). Those WOCs feature a significantly different ligand framework compared to the cubanes presented above. In contrast to the Co^III^ cubanes where the bridging oxo-ligands might be protonated and potentially participate in the O−O formation, such involvement is not possible in the case of the Co^II^ cubanes, since they are an essential part of the bidentate alkoxide ligands forming [${\text{Co}}_{4}^{\text{II}}$(hmp)_4_(μ−OAc)_2_(μ^2^-OAc)_2_(OH_2_)_2_] (hmp = 2-(hydroxymethyl)-pyridine) {Co4(OR)4} (Schilling et al., 2017). Those ligands turned out to be quite flexible which even allowed the incorporation of a redox-inert lanthanide cation into the cubane cage [${\text{Co}}_{3}^{\text{II}}$Ln(hmp)_4_(OAc)_5_(OH_2_)] (Ln = Ho-Yb, hmp = 2-(hydroxymethyl)pyridine), thereby closely mimicking nature's OEC containing the redox-inert calcium-ion (Evangelisti et al., 2015). The mechanism of [${\text{Co}}_{4}^{\text{II}}$(hmp)_4_(μ−OAc)_2_(μ^2^-OAc)_2_(OH_2_)_2_] was studied by Hodel and Luber (2016b), taking care of solvation effects by including the first solvation shell obtained from DFT-MD consisting of 68 H_2_O molecules into their model. The two most general mechanisms, namely a WNA and an i-RC were investigated in terms of their thermodynamics and kinetics. Both pathways are initiated by two consecutive PCETs, then they diverge and either a WNA or another two PCETs take place, before the O−O bond is formed via an i-RC reaction. The two pathways might be summarized as PCET-PCET-[WNA-PCET]-PCET-[O2DI-AQAS] and PCET-PCET-PCET-PCET-(i-RC)-[O2DI-AQAS]. Since the ligand environment was found to be stable, an i-RC mechanism, even though energetically similar to a WNA, appears to be less likely since it requires the association of another hydroxide or water molecule which was found to be energetically unfavorable. Nevertheless, an alternative pathway, i.e., a WNA on the bis-oxo species (Co^IV^ = O)_2_, was modeled since the localization of the lowest unoccupied molecular orbital (LUMO) indicated the electrophilicity of those oxo-ligands, which however was found to be inferior to the pathways discussed earlier. The in-depth study of the WNA pathways suggests that tuning of the LUMO energetics might give access to rational design of WOCs (Schilling et al., 2016). Similar WNA pathways were proposed for some mono-nuclear WOCs, all of which have in common that the highest formal oxidation state is Co^IV^ rather than Co^V^ (Wasylenko et al., 2011; Younus et al., 2017). Inclusion of a full first solvation shell allowed to observe, how the proton of the attacking H_2_O is transfered through a complex hydrogen bonding network to a H_2_O far away of the active site. Since there is no proton acceptor present, a high energy H_3_O^+^ is formed, which renders the WNA energetically unfavorable. In reality, those WOCs are however most active under slightly basic conditions.

**Figure 12:**
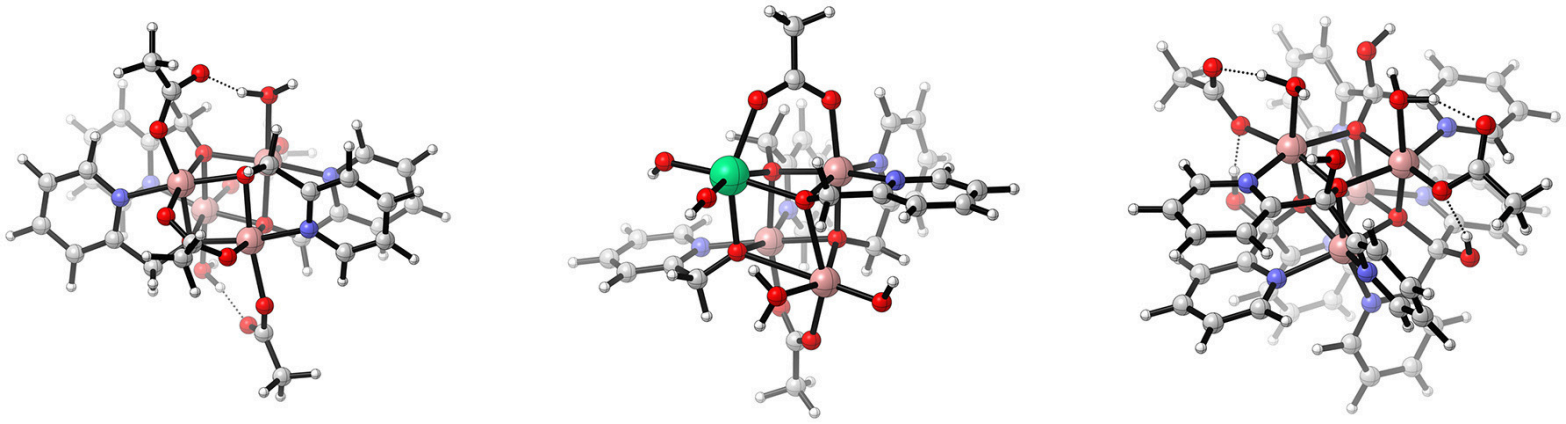
Schematic illustration of the cubanes studied by *Patzke* and co-workers: {Co_4_(OR)_4_} (**left**, Evangelisti et al. (2013)) and {Co3Ln(OR)4} (**middle**, Evangelisti et al. (2015)) (R = hmp, and Ln = Er, Tm, Yb, Ho), and the recently published third generation [${\text{Co}}_{4}^{\text{III}}$((dpy(OH)(O))_4_)(OAc)_2_(OH_2_)_2_]^2+^ (**right**, Song et al. (2017)). Extension to the color code: *lime* – erbium, thulium, ytterbium or holmium. Reproduced from geometrical data published by Evangelisti et al. (2013) **(left)** Evangelisti et al. (2015) **(middle)**, and Song et al. (2017) **(right)**.

**Figure 13:**
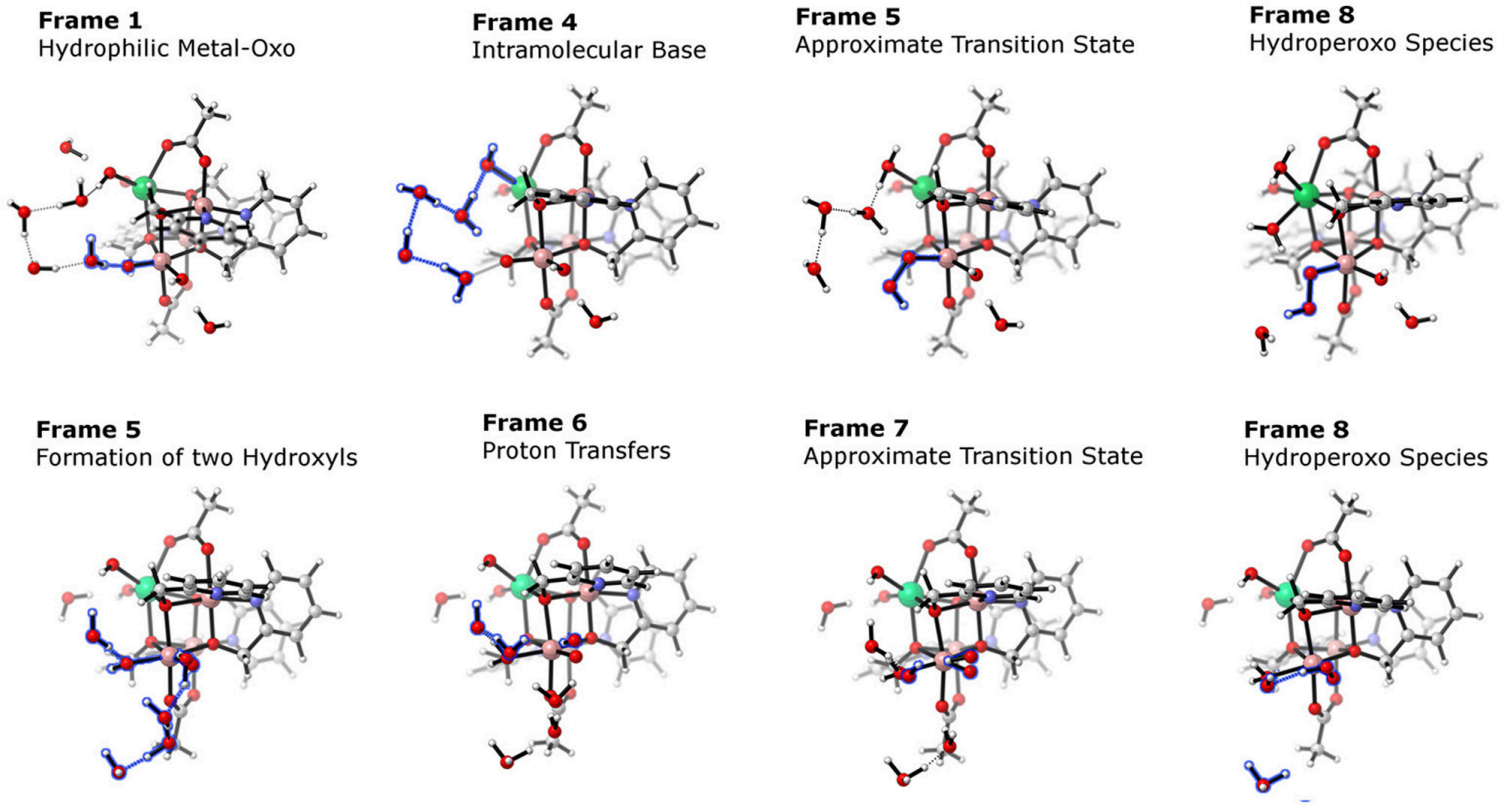
Approximate minimum energy pathways obtained by NEB optimizations for a WNA on two isomers of [${\text{Co}}_{3}^{\text{III}}$Er(hmp)_4_(μ^2^-OAc)_2_(OH)_3_(O)]. **Top**: Oxo-ligand is on the same face of the cubane as the erbium ion, note how the hydroxyl ligand acts as proton acceptor. **Bottom**: Oxo-ligand is on the face as another cobalt center, here the hydrogen bonding network is unable to transfer the proton to the hydroxyl ligand, instead a hydronium is formed. Reproduced from geometrical data published by Hodel and Luber (2016a).

A similar study was conducted to elucidate the role of the redox-inert metal center in [${\text{Co}}_{3}^{\text{II}}$Ln(hmp)_4_(OAc)(OH)_4_OH_2_] (LnCo_3_(OR)_4_; Ln = Er, Tm) (Evangelisti et al., 2015; Hodel and Luber, 2016a) (see Figure 12). Again explicit solvation turned out to improve the description of the catalytic pathway. Hydrogen bonding is crucial not only for the O−O bond formation but also for the stabilization of the cubane structure. For the initial steps of the catalytic cycle, distorted cubane core structures were found where one of the active cobalt center is slightly pulled out of the cubane core. The latter is associated with an inversion of the energetic ordering of the α-highest occupied molecular orbital (HOMO) and α-LUMO, which acts as an accepting orbital for the WNA, and thus increases the barrier for the O−O formation in the case of {Co_3_Tm(OR)_4_}. The different alignments of the ligands compared to the {Co_4_(OR)_4_} cubane only allowed a single-site mechanism, unless acetate bridges were substituted by aqua or hydroxyl ligands. The active cobalt center bears a hydroxyl and an aqua ligand in *cis*-orientation, thus there are two possible oxyl-species which can undergo a WNA (see Figure 13). The NEB calculations for the two possibles pathways again highlight the importance of the solvation shell. In one case the hydrogen bonding network is able to transfer the proton from the nucleophile to a hydroxyl ligand of the lanthanide which acts as an intramolecular base. In another instance, the solvation shell stabilizes a H_3_O^+^ and a OH^−^ molecule prior to the bond formation due to hydrogen bonding. Those findings illustrate the importance of explicit solvation but also the difficulties which arise from them. Extended hydrogen bonding networks are by virtue highly dynamic, static calculations are as a consequence a limited tool to capture the full picture.

**Figure 14:**
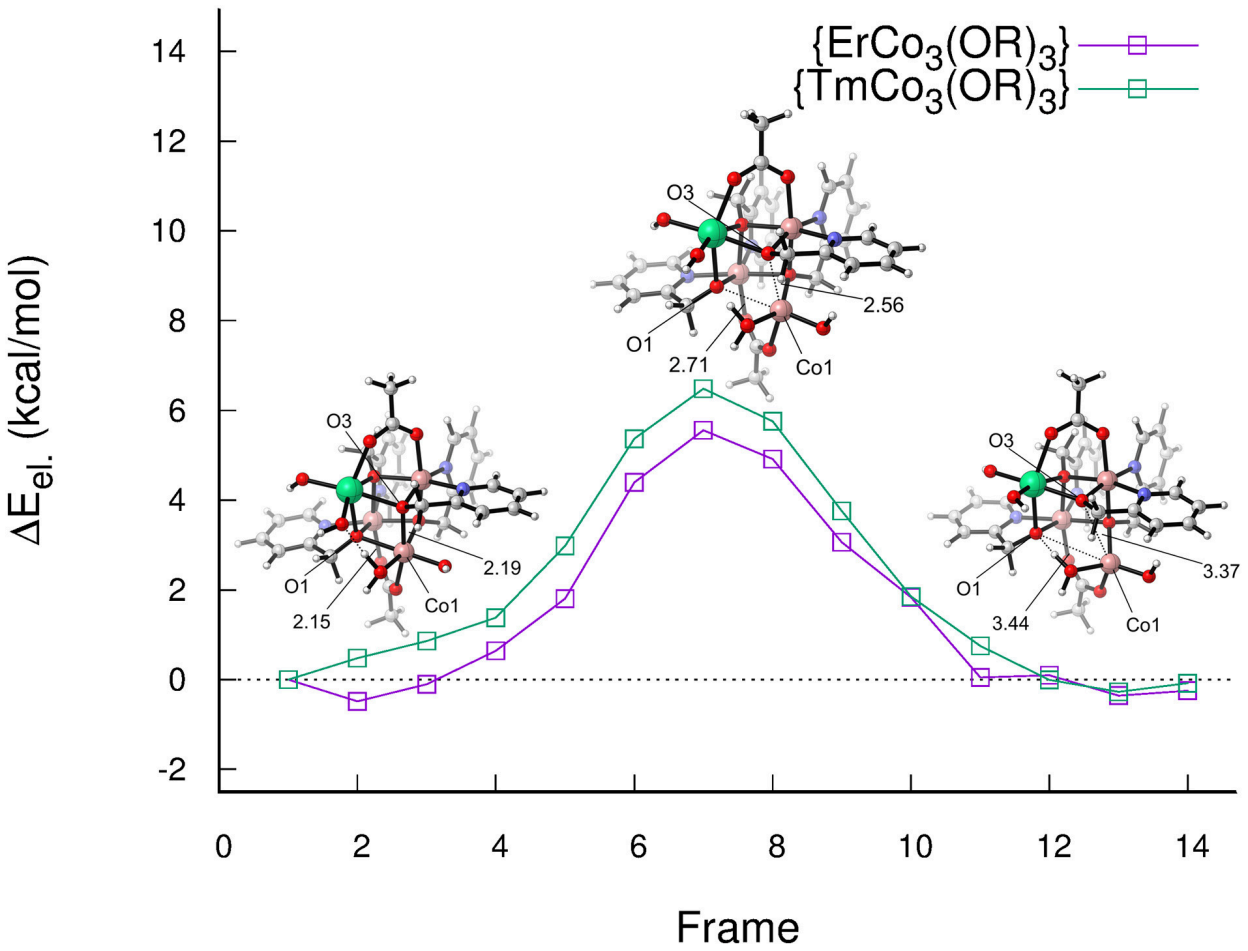
Approximate reaction path for the opening of the cubane core of {LnCo_3_(OR)_4_}, Ln = Er, Tm. Schilling et al. (2017), Copyright Wiley-VCH Verlag GmbH & Co. KGaA. Reproduced with permission.

In a follow up study Schilling et al. (2017) investigated the “distorted” cubane structures in more detail. It turned out that distorted structures are intermediates toward fully open cubane core structures (see Figure 14). The opening of the cubane cage is reminiscent of findings for the OCE where the dangling manganese is known to “pull-out” one corner of the cubane core (Krewald et al., 2015). This observation marks the first occurrence of an artificial cubane structure with similar structural flexibility. While approximate reaction barriers for the opening of the cubane core turned out to be rather small, the open structures barely offer an energetic advantage over a closed pathway in terms of thermodynamics. Even though the open structures were found to be stable when employing both implicit and explicit solvation there were particularly vast differences in the energetics, caused primarily by differences in the hydrogen-bonding network (i.e., the orientation of certain water molecules) when using an explicit solvation shell.

Not long ago the third generation of Co^II^ cubanes was presented by Song et al. (2017), which features two aqua ligands on the same face of the cube mimicking the surface of Co_*x*_O_3_. While the mechanism for water oxidation catalyzed by [${\text{Co}}_{4}^{\text{II}}$ ((dpy(OH)(O))_4_)(OAc)_2_(OH_2_)_2_]^2+^ (dpy(OH)(O) = di-2-pyridinylmethanediolat) is still under investigation, unprecedented new insight into the hydrogen bonding network of the active face of the cubane cubane were obtained using DFT-MD calculations. Song et al. (2017) also have shown experimentally that it is possible substitute some of the cobalt centers by Ni^II^. However, they were not able to isolate cubanes with a specific substitution pattern, but solid evidence comes from mass spectrometry that statistical substitution takes place. Doping of heterogeneous cobalt-oxides with nickel-ions has already been frequently used however not for homogeneous catalysts. Whether those mixed metal cubanes can help to identify the mechanism governing water oxidation is still under investigation.

**Figure 15:**
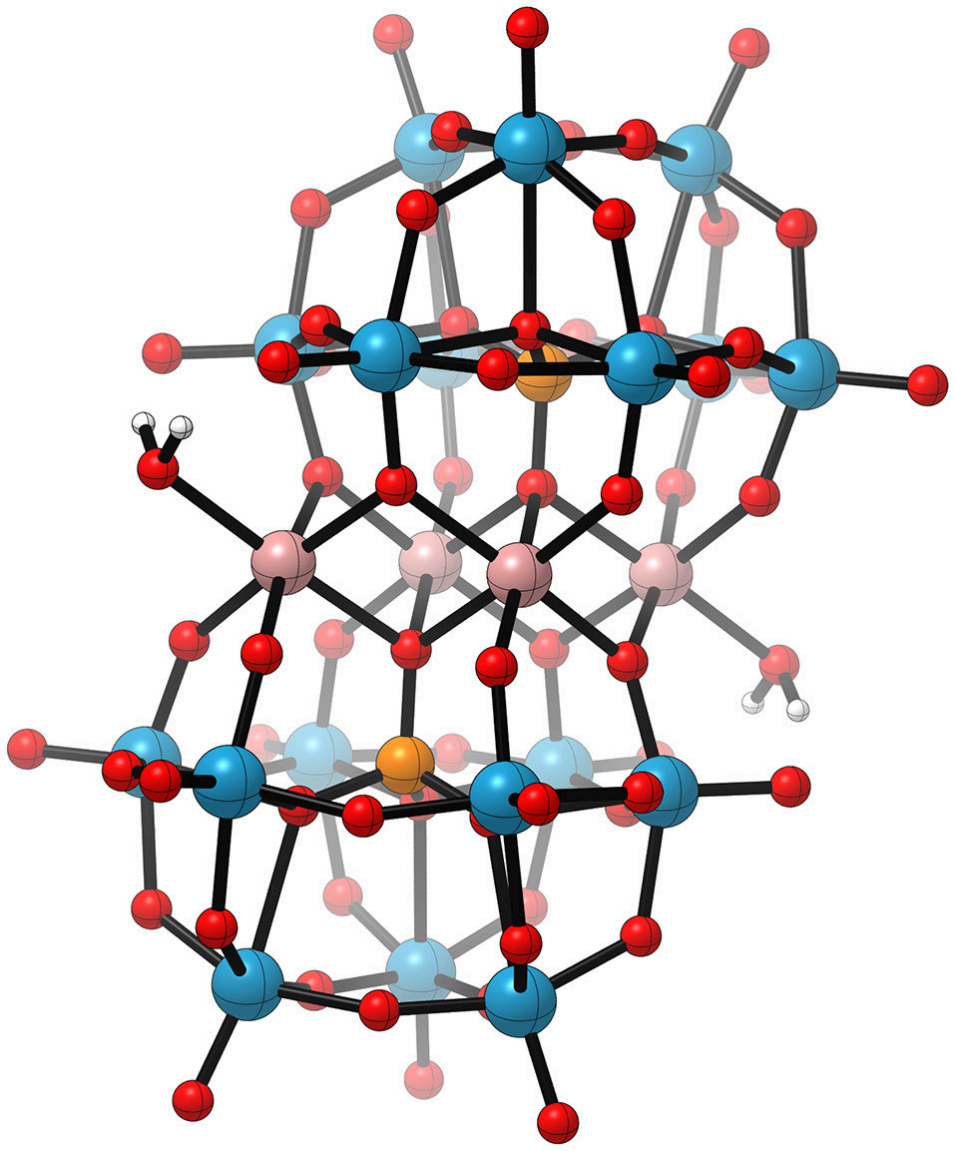
Schematic illustration of [Co_4_(H_2_O)_2_(XW_9_O_34_)_2_]^10−^ (X = P or V) POM studied by Soriano-López et al. (2017). Extension of the color code: *orange*–phosphorous or vanadium, *pale blue*-tungsten. Reproduced from geometrical data published by Soriano-López et al. (2017).

Besides the organometallic cubane clusters, there are also carbon-free Co^II^ WOCs, such as the polyoxometalates (POMs) presented by Soriano-López et al. (2017) Those complexes are composed of a tetra-cobalt oxide core which is sandwiched by two lacunary polytungstate cages [XW_9_O_34_ (X = P or V)] forming [Co_4_(OH_2_)_2_(XW_9_O_34_)_2_]^10−^ (see Figure 15). The cobalt atoms do not form a cuboidal core but rather a mimic of a cobalt oxide layer. As a consequence, only the two terminal metal centers possess aqua ligands, which limits O−O bond formation to inter – rather than intramolecular pathways. Since a bimolecular pathway seems unlikely due to the sheer size of the POM, only one option is left: PT-ET-PCET-[WNA-(i-PT)-ET]-PCET-[O2DI-AQAS] and concerted variations of it. Free energy calculations favored the sequence stated before which is identical to the mechanism proposed for the Co^II^-cubanes. The only difference is the capability of bridging oxo moieties (Co−O−W) to act as a proton acceptor, and thereby facilitate a WNA.

## 5. Summary and conclusion

In the following some general conclusions on the calculated mechanisms are presented. We want to emphasize that the goal of this summary is not to judge whether the proposed mechanisms are in agreement with experiments or not. The purpose is mere to compare the mechanisms from a theoretical point of view. Comparing the proposed mechanisms for a certain type of ligand framework and nuclearity, we find some correlations that might help to elucidate the reaction mechanism of yet undiscovered catalysts. However, there might also be the chance that up to now the proposed mechanisms are somewhat biased by the previous studies and in particular by the computational protocol applied.

There is general agreement on the first steps of the mechanism of mononuclear cobalt WOCs, which are supposed to undergo two PCETs before the O−O bond is formed by a WNA. Depending on the WOCs initial oxidation state, two electron transfers lead to either a formal Co^IV^ or Co^V^. All potential Co^V^ complexes are bearing non-innocent ligands which potentially are oxidized instead of the metal center, hence a formal oxidation state of Co^IV^ might be a sufficient requirement for water oxidation. Further, for the discussed mononuclear catalysts a bimolecular RC mechanism (I2M) was either excluded by experiments or rendered unlikely due to the strict octahedral coordination mode of the cobalt center as well as the sterically demanding ligands. An extension of the coordination sphere as observed for certain ruthenium-based catalysts seems unlikely for the used ligand-frameworks, which are rather confined in their flexibility.

The picture is similar for dinuclear catalysts in terms of the catalytically active species and formal oxidation state (Co^IV^ = O). However, when it comes to the O−O bond formation, i-RC becomes a respectable alternative to a WNA, since bulky ligands are hardly the limiting factor for intramolecular reactions. Regarding the Co^III^ cubodial model systems there is consent among all authors: two PCETs are followed by the O−O bond formation. The preferred mechanism for the latter depends on the protonation state (i.e., protonated μ–O, oxo-ligands on the same face of the cubane etc.) which in turn is dependent on the solvation model. The proposed mechanism for the *Dismukes-Cubane* changed slightly over the past years: from a WNA, over a WNA(OH), to an i-WNA(OH). The reason for the changes are mainly routed in the uncertainty about the exact structure (i.e., substitution of OAc-ligands by H_2_O or OH^−^) as well as the protonation state of the species in solution. Nevertheless, in general the mechanism is still consistent with the ones proposed for model systems. This is also true for the Co^II^-cubanes, which both supposedly follow a reaction scheme analogous to the one proposed for Co^III^ model systems. However, also here the authors have explored a variety of structures depending on which and how many of the OAc-ligands were exchanged. The non-cubodial cobalt-core of the Co^II^-POM was found to follow a similar water oxidation mechanism as the Co^II^-cubanes, since all those catalysts have in common that the number of metal centers accessible by solvent is limited.

From all those studies we can draw guidelines how to approach mechanistic studies of novel catalysts and how to improve current computational approaches. Of uttermost importance is the choice of an appropriate model system. Since the reaction takes place in the condensed phase, special attention has to be given to possible ligand substitutions and protonation state(s). The latter is in particular dependent on the experimental conditions, where the pH usually is controlled by buffers. But not only the catalyst and buffer ions are part of the reaction solution, there are also counter ions (e.g., of the catalyst or the oxidant) and chemical oxidants as, in the case of photocatalytic water oxidation, photosensitzers and sacrificial electron acceptors. If and how those other molecules influence the reaction mechanism is often not directly obvious and might be worth to be investigated.

Regarding the catalyst itself, for most of them solvent-solute interaction are non-negligible. Besides static calculations, which are biased to the initial guess used in the calculations, AIMD approaches, although computationally more expensive, can lead to a more complete picture of the catalytic process. If one attempts to identify the rate limiting step in terms of an activation barrier, one has to keep in mind that there are other potentially rate limiting reactions besides the O−O bond formation. Hence, an appropriate solvation model has to be chosen for the modeling of thermodynamics and reaction barriers. So far, there is no gold standard which can be routinely applied to all kinds of systems, which makes the choice crucial. When it comes to modeling the electronic structure of the catalyst, special care has to be given to potentially non-innocent ligands, metal-oxo species as well as to metal-metal interactions in the case of poly-nuclear WOCs. In this context, multiconfigurational methods might help to overcome the limitations of DFT. However, often the complexity and size of the WOCs does not allow for a sufficiently large active space in multiconfigurational calculations, which currently limits their application to rather small systems. Nevertheless, the necessity to use such methods is for example illustrated by O−O bond formation, for which a M^IV^=O or M^V^=O species is a prerequisite. The accurate description of the latter is a rather difficult task due to the open-shell nature of both the metal center and the oxo ligand. Another issue are formal oxidation states, since this chemical concept has no strict counter part in quantum mechanics, and thus may lead to misunderstandings among theoreticians and between theoreticians and experimentalists.

## Author contributions

The authors MS and SL jointly developed the concept of the review and wrote the manuscript. All shown structures were plotted using coordinates of optimized structures provided by the authors of the corresponding publications or if not available, where rebuild without optimization according to figures in the manuscripts, visualized using CYLView which is developed by Legault (2008-2018).

### Conflict of interest statement

The authors declare that the research was conducted in the absence of any commercial or financial relationships that could be construed as a potential conflict of interest.
